# Heterometallic 3d–4f Alkoxide Precursors for the Synthesis of Binary Oxide Nanomaterials

**DOI:** 10.1021/acs.inorgchem.2c03872

**Published:** 2023-01-25

**Authors:** Rafał Petrus, Adrian Kowaliński, Józef Utko, Karolina Matuszak, Tadeusz Lis, Piotr Sobota

**Affiliations:** † Faculty of Chemistry, Wrocław University of Science and Technology, 23 Smoluchowskiego, 50-370 Wrocław, Poland; ‡ Faculty of Chemistry, University of Wrocław, 14 F. Joliot-Curie, 50-383 Wrocław, Poland

## Abstract

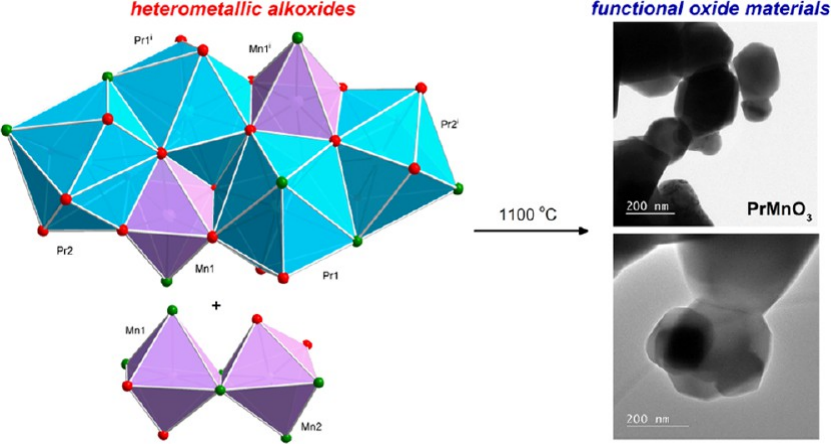

In this study, a new method for the synthesis of heterometallic 3d–4f alkoxides by the direct reaction of metallic lanthanides (La, Pr, Nd, Gd) with MCl_2_ (M = Mn, Ni, Co) in 2-methoxyethanol was developed. The method was applied to the synthesis of the heterometallic oxo-alkoxide clusters [Ln_4_Mn_2_(μ_6_-O)(μ_3_-OR)_8_(HOR)_*x*_Cl_6_] (Ln = La (**1**), Nd (**2**), Gd (**3**); *x* = 0, 2, 4); [Pr_4_M_2_(μ_6_-O)(μ_3_-OR)_8_(HOR)*_x_*Cl_6_] (M = Co (**4**), Ni (**5**); *x* = 2, 4); and [Ln_4_Mn_2_(μ_3_-OH)_2_(μ_3_-OR)_4_(μ-OR)_4_(μ-Cl)_2_(HOR)_4_Cl_6_] (Ln = La (**11**) and Pr (**12**)). Mechanistic investigation led to the isolation of the homo- and heterometallic intermediates [Pr(μ-OR)(μ-Cl)(HOR)Cl]*_n_* (**6**), [Co_4_(μ_3_-OR)_4_(HOR)_4_Cl_4_] (**7**), [Ni_4_(μ_3_-OR)_4_(HOEt)_4_Cl_4_] (**8**), [Mn_4_(μ_3_-OR)_4_(HOR)_2_(HOEt)_2_Cl_4_] (**9**), and [Nd(HOR)_4_Cl][CoCl_4_] (**10**). In the presence of an external M(II) source at 1100 °C, **1**–**4** and **12** were selectively converted into binary metal oxide nanomaterials with trigonal or orthorhombic perovskite structures, i.e., LaMnO_3_, GdMnO_3_, NdMnO_3_, Pr_0.9_MnO_3_, and PrCoO_3_. Compound **5** decomposed into a mixture of homo- and heterometallic oxides. The method presented provides a valuable platform for the preparation of advanced heterometallic oxide materials with promising magnetic, luminescence, and/or catalytic applications.

## Abstract 2

In this study, an efficient procedure for the synthesis of a family of heterometallic 3d−4f alkoxides was developed, providing crystalline precursors for obtaining binary metal oxide materials. The selective formation of heterometallic, industrially important, functional nanomaterials with trigonal or orthorhombic perovskite structures, *i.e*., LaMnO_3_, GdMnO_3_, NdMnO_3_, Pr_0.9_MnO_3_, and PrCoO_3_, was reported.

## Introduction

Over the last three decades, heterometallic 3d–4f complexes have attracted much attention because of their structural diversity, luminescence, and magnetic or adsorptive properties. Of particular interest are coordination compounds that behave as single-molecule magnets (SMMs) because of their potential applications in high-density information storage, spintronic devices, and quantum computation.^1^ Because of their large single-ion anisotropy, lanthanide complexes show great potential for SMMs. However, their combination with 3d metal ions is essential to effectively suppress quantum tunneling and establish magnetic exchange between metal ions (3d–4f).^2−4^ The first example of a 3d–4f SMM was [CuTb(hfac)_2_(L)]_2_ (H_3_L = 1-(2-hydroxybenzamido)-2-(2-hydroxy-3-methoxy-benzylideneamino)-ethane; hfacH = hexafluoroacetylacetone), which has an effective energy barrier of 21 K, a relaxation time of 2.7 × 10^–8^ s, and an estimated blocking temperature of 1.2 K.^5^ Since this breakthrough, combinations of Tb(III), Dy(III), Er(III), Sm(III), Yb(III), Gd(III), or Ho(III) with transition-metal ions such as Co(II), Mn(II/III), Fe(II/III), Ni(II), and Cu(II) have been extensively studied.^6^

Other than the above examples of SMMs, the application of heterometallic 3d–4f clusters is limited. Nevertheless, their multiple adjacent metal sites together with their unique electronic and structural features offer the promise of catalytic performance. One of the few examples of such catalytic application is that of [Gd_102_Ni_36_(μ_3_-OH)_132_(L^2^)_18_(L^3^)_18_(H_2_L^3^)_24_(OAc)_84_(SO_4_)_18_(NO_3_)_18_(H_2_O)_30_]Br_6_,(NO_3_)_6_ (Gd_102_Ni_36_, HL^2^ = 2-mercapto-5-methyl-1,3,4-thiadiazide; H_3_L^3^ = 2,2-dimethylol propionic acid), which shows remarkable activity in photocatalytic CO_2_ reduction.^7^ Another catalytic application of these compounds is as electrocatalysts for the splitting of water into H_2_ and O_2_. For example, [Eu_36_Co_12_(μ_4_-O)_6_(μ_3_-OH)_84_(OAc)_18_(H_2_O)_42_(NO_3_)_7_Cl_2_]^9+^ shows effective water oxidation activity under acidic conditions, owing to the synergistic effect of Eu(III) and Co(II) ions on O–O bond formation.^8^ Heterometallic cooperativity in water oxidation has also been reported for [Ln_2_Mn_2_(O_2_CMe)_6_(pdmH)_2_(L)](NO_3_) (Ln = Dy and Gd; pdmH_2_ = 2,6-pyridine dimethanol; H_2_L = (6-hydroxymethylpyridin-2-yl)-(6-hydroxymethylpyridin-2-ylmethoxy-methanol)).^9^ The series [LnCo_3_(hmp)_4_(OAc)_5_(H_2_O)], where Ln = Ho, Er, Tm, or Yb (hmpH = 2-(hydroxymethyl)pyridine),^10^ and [NdCo_3_(btp)_2_(OAc)_2_(NO_3_)_2_](NO_3_) (btp = 2,6-bis(1,2,3-triazol-4-yl)pyridine) anchored in phospho-doped graphitic carbon nitride,^11^ are further examples of water oxidation catalysts, being mimetics of the {CaMn_4_O_5_} oxygen evolution complex in photosystem II. [Ln_52_Ni_56_(IDA)_48_(OH)_154_(H_2_O)_38_]^18+^ clusters (Ln = Pr, Eu, Gd; IDA = iminodiacetate) supported on CdS forms lanthanide–transition-metal catalysts Ln_52_Ni_56-***x***_Cd_***x***_/CdS that show high photocatalytic efficiency for hydrogen evolution.^12^ Furthermore, the iodide-templated three-dimensional (3D)-coordination polymer {[Eu_2_Cu_5_(OH)_2_(pydc)_6_(H_2_O)_8_]·I_8_}*_n_* (pydc = pyridine-2,6-dicarboxylate) has been demonstrated to be an efficient photocatalyst for H_2_ production under UV irradiation.^13^ Heterometallic lanthanide–zinc clusters have also been frequently used as catalysts for C–C, C–N, or C–O bond formation by reacting CO_2_ with terminal alkynes, aziridines, or epoxides.^14,15^ Another interesting example of heterometallic 3d–4f compounds are one-dimensional (1D) or 3D-coordination polymers constructed from 3,5-pyrazoledicarboxylate (pdc^3–^) bridged Cu^II^Ln^III^ dinuclear units and sulfate anions of {[CuLn_2_(pdc)_2_(SO_4_)(H_2_O)_6_]·H_2_O}*_n_* (Ln = Tb, Dy) and {[CuLn_2_(pdc)_2_(SO_4_)(H_2_O)_4_]·H_2_O}*_n_* (Ln = Sm, Eu, Gd, Tb, Dy).^16^ 3d–4f metal-based 1D ladder-like polymer {[Yb(pydc)_3_Mn_1.5_(H_2_O)_6_]·6H_2_O}*_n_* was the first example of coordination compounds in which a decrease in the amount of coordinated water molecules on the Mn(II) ion led to transformation into 3D zeolite-type complex {[Yb(pydc)_3_Mn_1.5_(H_2_O)_3_]·1.5H_2_O}*_n_*.^17^ The 3D polymers {[Ln(pydc)_3_Mn_1.5_(H_2_O)_3_]·3.25H_2_O}*_n_* (Ln = Eu, Tb) with high-performance selectivity for Zn(II) are promising luminescent probes based on a 3d–4f coordination network.^18^ {[Tb_2_(Cu_8_I_8_)(pba)_6_(H_2_O)_4_]·C_4_H_8_O_2_}*_n_* (pba^–^ = 3-(pyridin-4-yl)benzoate) was a 3d–4f luminescent metal–organic framework (MOF) that exhibits high recognition selectivity, and sensing capability originate from emissions of the dual luminescent centers.^19^ {[(CH_3_)_2_NH_2_]_2_[Zn_2_Ln_2_(fda)_6_(DMF)_2_]·2DMF}*_n_* (Ln = Eu, Tb; fda^2–^ = furan-2,5-dicarboxylate) MOFs can serve as luminescent sensors for the fast response and highly selective detection of aniline via luminescence quenching.^20^

The use of heterometallic 3d–4f compounds as single-source molecular precursors for the low-temperature synthesis of functional inorganic materials is also very limited. One of the few examples is the series of isostructural compounds [Fe_2_Ln_2_((OCH_2_)_3_CR)_2_(O_2_C*^t^*Bu)_6_(H_2_O)_4_] (Ln = La, Gd and R = Me, Et), which were used to prepare lanthanide orthoferrite perovskites (LnFeO_3_).^21^ Another example is the use of an equimolar mixture of (NH_4_)[Ln(EDTA)] (Ln = Pr, Sm, Eu, Gd, Dy, Er) and (NH_4_)_3_[V(O)_2_(EDTA)] at 800 °C for the preparation of lanthanide vanadates (LnVO_4_).^22^ Furthermore, the solid-phase thermal decomposition of [Ln(Mn(CO)_3_Cp^COOH^)_2_(OAc)(MeOH)]*_n_* (Ln = Nd, Gd, Dy), [Ln_2_(Mn(CO)_3_Cp^COOH^)_4_(OAc)_2_(H_2_O)_4_] (Ln = Ho, Er, Tm), and [Ln_2_(Mn(CO)_3_Cp^COOH^)_4_(NO_3_)_2_(DME)_2_] (Ln = Eu, Tb) at 670–900 °C has been investigated for the synthesis of metamagnetic LnMn_2_O_5_.^23,24^

Recently, we reported a method for the synthesis of functional nanomaterials, i.e., La_0.66_TiO_3_, Nd_0.66_TiO_3_, La_2_Zr_2_O_7_, La_2_Hf_2_O_7_, Nd_2_Zr_2_O_7_, and Nd_2_Hf_2_O_7_, by thermolysis of the group-4 lanthanide oxo-alkoxides [Ln_2_Ti_4_(μ_4_-O)_2_(μ_3_-OEt)_2_(μ-OEt)_8_(OEt)_6_(HOEt)_2_Cl_2_] (Ln = La, Nd); [La_2_M′_2_(μ_3_-O)(μ-OEt)_5_(μ-Cl)(OEt)_2_(HOEt)_4_Cl_4_]_*n*_; and [Nd_4_M′_4_(μ_3_-O)_2_(μ-OEt)_10_(μ-Cl)_4_(OEt)_8_(HOEt)_10_Cl_2_] (M′ = Zr, Hf).^25^

The heterometallic cooperativity of 3d and 4f ions in transition-metal–lanthanide clusters can enhance their magnetic, luminescence, and/or catalytic properties. One of the main challenges faced in the rational design of 3d–4f complexes is that 3d and 4f metal ions have different chemical properties, coordination capabilities, and ionic radii. For the construction of 3d–4f clusters, we used 2-methoxyethanol as a ligand because it tends to form heterometallic compounds based on the hexanuclear M_6_(μ_6_-O) motif. The synthesis of 3d–4f complexes was performed using an uncommon synthetic method involving the direct reaction of metallic lanthanides (Ln = La, Pr, Nd, Gd) with divalent transition-metal chlorides (MCl_2_, where M(II) = Mn(II), Ni(II), or Co(II)) in a 2-methoxyethanol solution. This approach led to the synthesis of the new heterometallic alkoxide clusters [Ln_4_Mn_2_(μ_6_-O)(μ_3_-OR)_8_(HOR)_*x*_Cl_6_] (Ln = La (**1**), Nd (**2**), Gd (**3**); *x* = 0, 2, 4); [Pr_4_M_2_(μ_6_-O)(μ_3_-OR)_8_(HOR)_*x*_Cl_6_] (M = Co (**4**), Ni (**5**), and *x* = 2, 4); and [Ln_4_Mn_2_(μ_3_-OH)_2_(μ_3_-OR)_4_(μ-OR)_4_(μ-Cl)_2_(HOR)_4_Cl_6_] (Ln = La (**11**) and Pr (**12**)), which were converted to binary metal oxide materials.

## Results and Discussion

### Syntheses and Structural Study

**Scheme 1:**
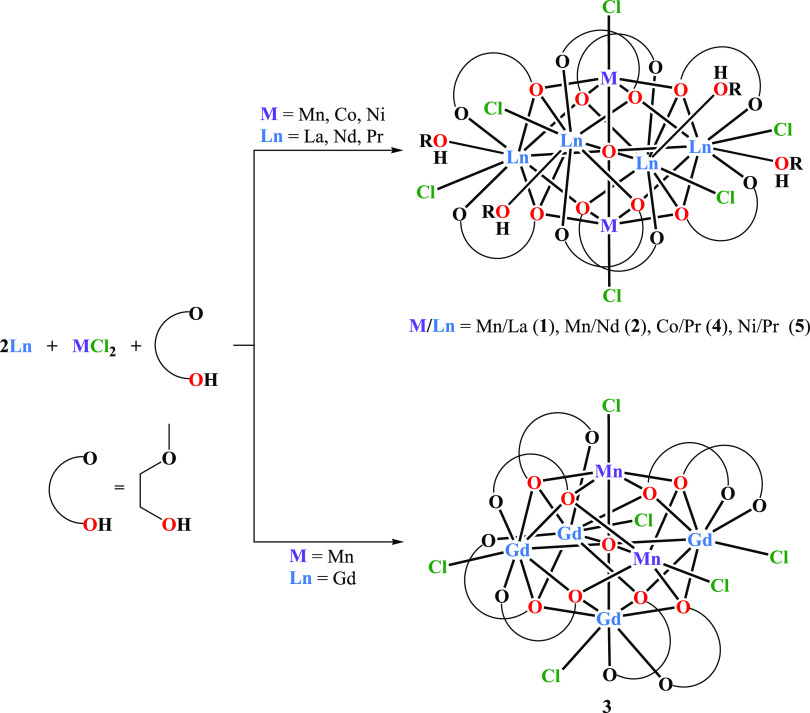
Synthesis of **1**–**5**

The reaction of 2 equiv metallic La, Pr, Nd, or Gd with 1 equiv MCl_2_ (M(II) = Mn(II), Ni(II), or Co(II)) in 2-methoxyethanol gave the crystalline 3d–4f oxo-alkoxide clusters [La_4_Mn_2_(μ_6_-O)(μ_3_-OR)_8_(HOR)_4_Cl_6_] (**1**, 46%), [Nd_4_Mn_2_(μ_6_-O)(μ_3_-OR)_8_(HOR)_2_Cl_6_] (**2**, 51%), [Gd_4_Mn_2_(μ_6_-O)(μ_3_-OR)_8_Cl_6_] (**3**, 46%), [Pr_4_Co_2_(μ_6_-O)(μ_3_-OR)_8_(HOR)_2_Cl_6_] (**4**, 47%), and [Pr_4_Ni_2_(μ_6_-O)(μ_3_-OR)_8_(HOR)_4_Cl_6_] (**5**, 44%), (Scheme 1). The direct route for the lanthanide compounds from the pure metals has not been considered attractive because it usually requires activation by mercury or iodine, prolonged heating, and the use of heavy-metal reagents or mercury diaryls.^26,27^

**Figure 1:**
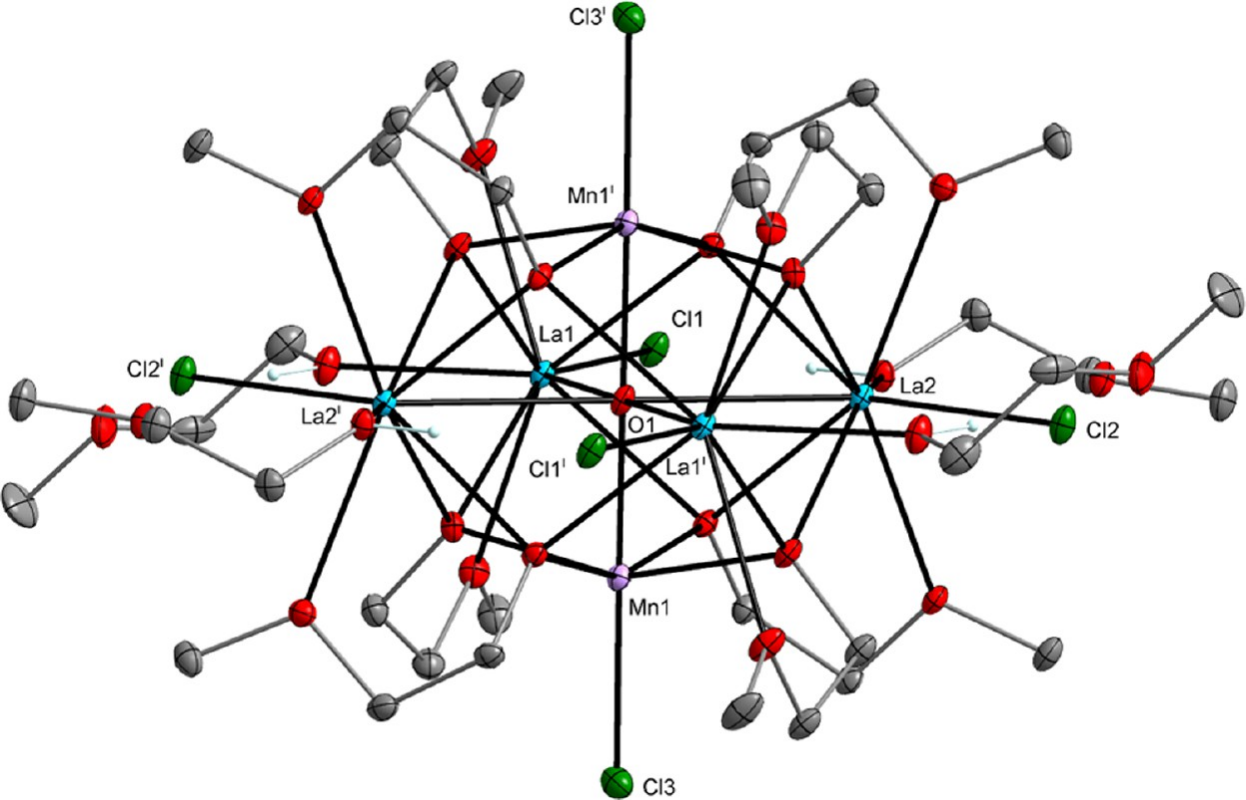
Molecular structure of [La_4_Mn_2_(μ_6_-O)(μ_3_-OR)_8_(HOR)_4_Cl_6_] (**1**), which is isostructural with [Pr_4_Ni_2_(μ_6_-O)(μ_3_-OR)_8_(HOR)_4_Cl_6_] (**5**) (SI, Figure S1). Displacement ellipsoids are drawn at the 20% probability level. Hydrogen atoms of the alkyl groups, solvent molecules, and the second part of the disordered alkoxy ligands are omitted for clarity [symmetry code: (i) −*x*+1, −*y*+1, −*z*+1].

**Figure 2:**
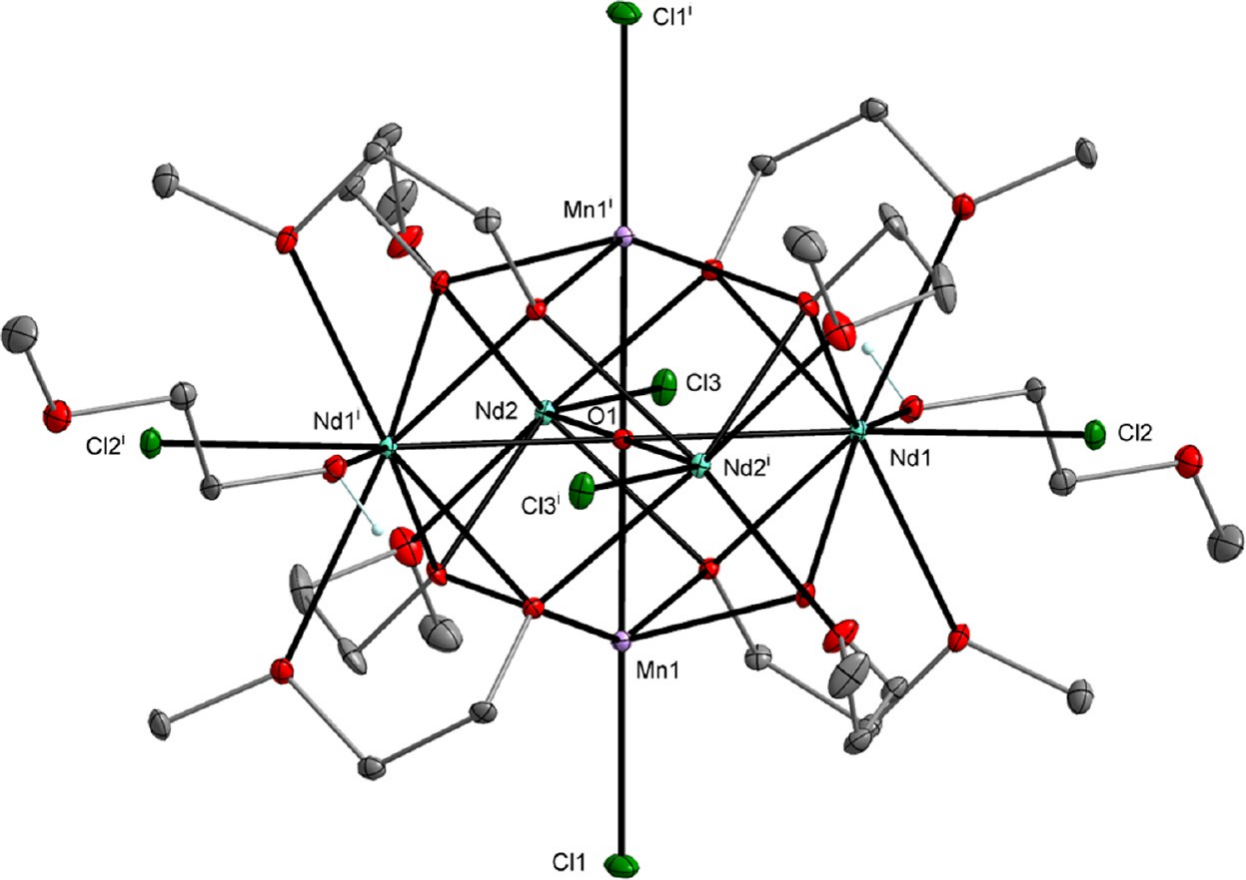
Molecular structure of [Nd_4_Mn_2_(μ_6_-O)(μ_3_-OR)_8_(HOR)_2_Cl_6_] (**2**), which is isostructural with [Pr_4_Co_2_(μ_6_-O)(μ_3_-OR)_8_(HOR)_2_Cl_6_] (**4**) (SI, Figure S2). Displacement ellipsoids are drawn at the 20% probability level. Hydrogen atoms of the alkyl groups, solvent molecules, and the second part of the disordered alkoxy ligands are omitted for clarity [symmetry code: (i) −*x*+1, −*y*+1, −*z*+1].

**Figure 3:**
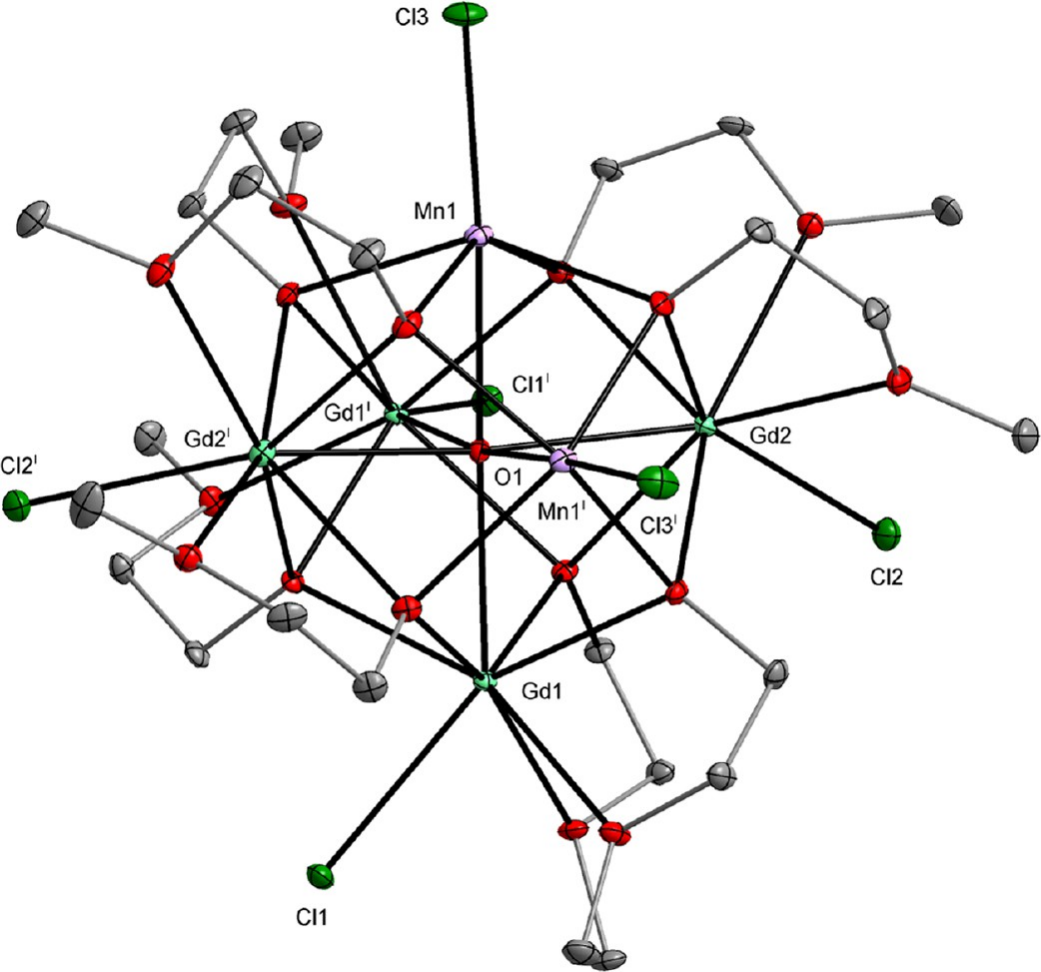
Molecular structure of [Gd_4_Mn_2_(μ_6_-O)(μ_3_-OR)_8_Cl_6_] (**3**). Displacement ellipsoids are drawn at the 30% probability level. The hydrogen atoms of the alkyl groups and the second part of the disordered alkoxy ligands are omitted for clarity [symmetry code: (i) −*x*+1, −*y*+1/2, *z*].

The single-crystal X-ray diffraction (XRD) analysis revealed that **1**–**5** form heterometallic hexanuclear clusters with the general formula [Ln_4_M_2_(μ_6_-O)(μ_3_-OR)_8_(HOR)*_x_*Cl_6_], where *x* = 0, 2, or 4 as shown in Figures 1–3 (Supporting Information (SI), Figures S1 to S2). The central cores of these structures are mainly based on Ln_4_M_2_(μ_6_-O) octahedrons in which two M atoms occupy axial positions, four Ln atoms are in equatorial positions, and a μ_6_-O encapsulated oxygen atom resides at the center of the octahedron (**1**–**2** and **4**–**5**). This structural motif has been frequently observed for the group-4 alkaline earth chloro-alkoxides [M_4_M′_2_(μ_6_-O)(μ_3_-OR)_8_(OR)_2_(HOR)*_x_*Cl_6_], where M(II) = Sr(II) or Ba(II); M′(IV) = Ti(IV), Zr(IV), or Hf(IV); and *x* = 0, 2, or 4,^28−31^ while lanthanide 2-methoxyethoxides usually form clusters with higher nuclearity, i.e., [Dy_10_(μ-OR)_20_(OR)_10_],^32^ [Gd_6_(μ_4_-O)(μ_3_-OR)_4_(μ-OR)_8_(OR)_4_],^33^ [Pr_8_(μ_4_-O)_4_(μ_3_-OR)_8_(μ-OR)_8_(OPMe_3_)_2_],^34^ and [Eu_4_(μ_3_-OR)_4_(OR)_4_(HOAr)_4_] (ArOH = 2,6-dimethylphenol or 2,6-diisopropylphenol).^35^ Ln(III) ions have a high tendency to form oxo-alkoxides instead of homoleptic alkoxides. Therefore, the most probable sources of O^2–^ anion are ether elimination reactions rather than adventitious hydrolysis.^36^

Manganese 2-methoxyethoxide has been reported in the form of the tetranuclear compounds [Mn_4_(μ-OR)_6_X_4_] and [Mn_4_(μ_3_-OR)_4_(HOR/HOEt)_4_X_4_] (X = Cl, Br),^37,38^ as well as the octanuclear [Mn_8_(μ_3_-OR)_8_(μ-Cl)_2_(HOR)_6_Cl_6_],^39^ nonadecanuclear [Mn_19_(μ_3_-O)_12_(μ_3_-OR)_12_(μ-OR)_2_(μ-HOR)_10_],^40^ and undeca- or tetradecametalate ionic compounds [Mn_10_(μ_4_-O)_4_(μ_3_-Cl)_4_(μ-OR)_12_][MnCl_4_] and [Mn_10_(μ_4_-O)_4_(μ_3_-Cl)_4_(μ-OR)_12_][Mn_4_(μ-Cl)_2_(HOR)_4_Cl_6_], which are based on the same decamanganate cation [Mn_10_(μ_4_-O)_4_(μ_3_-Cl)_4_(μ-OR)_12_]^2+^.^41,42^ In **1**–**5**, divalent metal cations are surrounded by one chloro, four alkoxo, and one oxo oxygen atom, which form distorted octahedrons.

Continuous-shape measurement (CShM)^43^ of the coordination environment around the M(II) ions in **1**–**5** revealed comparable departures from ideal octahedral geometry for compounds **1**, **4**, and **5**, with their metric shape parameters *S*(Oh) equal to 1.802 for Mn(II), 1.655 for Ni(II), and from 1.854 to 1.902 for Co(II). The *S*(Oh) parameters of 2.621–2.749 and 2.989 for **2** and **3**, respectively, indicate more significant distortions of octahedrons formed by the Mn(II) ions. Lanthanide ions surrounded by O_8_Cl donors adopt capped square antiprism, gyroelongated square pyramid J10, or muffin geometries. Lanthanide ions surrounded by O_7_Cl donor atoms form triangular dodecahedron, biaugmented trigonal prism, Johnson biaugmented trigonal prism (J50), or triangular dodecahedron geometries, as verified by the shape measurement calculations (SI, Table S2).

In the structures of **1**–**5**, the M atoms usually occupy the axial positions of the M_2_Ln_4_(μ_6_-O) octahedron and form M–O–M^i^ angles of 180°. A partially different central core geometry is observed for compound **3**, which contains Mn atoms in axial and equatorial positions with Mn1–O–Mn1^i^ angles of 83.87(9)° and 88.87(7)° (Figure 3).

The Mn–O_(oxo)_, Mn–O_(alkoxo)_, and Mn–Cl bond lengths for **1**–**3**, i.e., 2.006(3)–2.288(3), 2.162(3)–2.290(2), and 2.319(2)–2.386(4) Å, respectively, are similar to those reported for [Mn_10_(μ_4_-O)_4_(μ_3_-I)_3_(μ_3_-OH)(μ-OR)_12_][I_3_]_2_,^44^ [Mn_7_(μ_3_-OMe)_6_(μ-OMe)_6_(dbm)_6_] (dbm = dibenzoylmethane),^45^ [Mn_4_(μ-Cl)_8_(HOR)_4_]*_n_*,^46^ and [Mn_4_(μ_3_-OMe)_4_(OMe)_4_(dpm)_4_] (dpm = dipivaloylmethane).^47^ For the lanthanide ions in **1**–**3**, the resulting metric parameters do not deviate significantly from the values observed within the investigated group of compounds and are particularly close to those observed for [La_3_(μ_3_-OR^1^)_2_(μ-OR^1^)_3_(OR^1^)_4_] (R^1^O^–^ = 1-methoxy-2-methylpropoxide),^48^ [La_2_(μ-Cl)_2_(HOMe)_8_Cl_4_],^49^ [La_2_(μ-OR^2^)_2_(HOR^2^)_4_Cl_4_] (R^2^O^–^ = 1-methoxypropoxide),^50^ [Nd_2_(μ-Cl)_2_(HO*^i^*Pr)_6_Cl_4_],^51^ [Nd_5_(μ_5_-O)(μ_3_-O*^i^*Pr)_2_(μ_2_-O*^i^*Pr)_6_(O*^i^*Pr)_5_(HO*^i^*Pr)_2_],^52^ [Gd_5_(μ_5_-O)(μ_3_-O*^i^*Pr)_4_(μ_2_-O*^i^*Pr)_4_(O*^i^*Pr)_5_(HO*^i^*Pr)_2_],^53^ and [GdCl_3_(THF)_3_].^54^ For the heterometallic compounds **4** and **5**, the bond lengths of M–O_(oxo)_ (for **4** and **5**: 2.012(2)–2.022(2) and 1.987(2) Å), M–O_(alkoxo)_ (2.150(9)–2.239(9) and 2.139(2)–2.143(2) Å), and M–Cl (2.301(4)–2.305(3) and 2.299(2) Å) agree well with the values observed for [Co_4_(μ_3_-OR^3^)_2_(μ-OR^3^)_4_Cl_2_] (R^3^O^–^ = 2-ethoxyethoxide),^55^ [Co_4_(μ_3_-OR^4^)_4_Cl_4_] (R^4^O^–^ = 2-(N-(2-hydroxyethyl)amino)ethoxide),^56^ [M_4_(μ_3_-hmp)_4_(HOMe)_4_(Cl)_4_] (M = Co, Ni),^57,58^ [M_5_Ti(μ_6_-O)(μ-acac)_6_(μ-OEt)_6_] (M = Co, Ni),^59,60^ [Ni_4_(μ_3_-OMe)_4_(acac)_4_(HOMe)_4_],^61^ [Ni(trop)_2_(HOMe)_2_] (trop = tropolonato),^62^ and [Ni_5_Sb_3_(μ_4_-O)_2_(μ_3_-OEt)_3_(μ-OEt)_9_(OEt)_3_(HOEt)_4_].^63^ The Pr–O_(oxo)_ (for **4** and **5**: 2.554(2)–2.684(2) and 2.600(2)–2.648(2) Å), Pr–O_(alkoxo)_ (2.447(8)–2.486(9) and 2.417(3)–2.475(3) Å), and Pr–Cl (2.756(4)–2.851(3) and 2.810(2)–2.851(2) Å) bond lengths are similar to those found in [Pr_4_(μ_4_-O)(μ-OMe)_6_(3-NO_2_Tp)_4_] (3-NO_2_Tp^–^ = 3-nitrotrispyrazolylborate),^64^ [Pr_2_(μ-OC(CF_3_)_2_CH_3_)_2_(OC(CF_3_)_2_CH_3_)_4_(NH_3_)_4_], [Pr_3_(μ_3_-OC(CF_3_)_2_CH_3_)_2_(μ_3_-OC(CF_3_)_2_CH_3_)_3_(OC(CF_3_)_2_CH_3_)_4_],^65^ [PrCl_3_(THF)_2_]*_n_*,^66^ and [PrCl_3_(*^n^*PrOH)_6_]*_n_*.^67^ In the field of structural chemistry, **4** and **5** were the first reported alkoxides of Co(II)/Ni(II) and Pr(III). We also observed that recrystallization of **4** from ethanol solution leads to the formation of [Pr_4_Co_2_(μ_6_-O)(μ_3_-OR)_8_(EtOH)_4_Cl_6_] (**4a**), which contains EtOH molecules coordinated to each of the Pr(III) ions (SI, Figure S3).

Moreover, examples of coordination compounds based on these elements are very limited. For example, only five Co(II/III)-Pr(III) complexes, i.e., [Pr_4_Co_4_(μ_3_-OR^5^)_2_(μ_3_-OH)_2_(μ-OR^5^)_2_(μ-piv)_8_(μ-N_3_)_2_(NO_3_)_2_] (piv = pivalato),^68^ [PrCo(μ-OAr)_2_(H_2_O)_2_(NO_3_)_4_] (ArO = 3-methoxy-2-oxo-benzaldehyde),^69^ [Pr_2_Co_3_(cmipa)_4_(H_2_O)_10_] (cmipa = 4-(carboxylatomethoxy)isophthalato),^70^ [Pr_2_Co_5_(μ_3_-OAr)_4_(μ_3_-OH)_4_(μ-ac)_2_(HNAr)_2_(NO_3_)_6_(MeCN)_4_] (ac = acetato, ArO = 6-chloropyridin-2-olato, HNAr = 6-chloropyridin-2-onato),^71^ and [PrCo(μ-ac)_2_(μ-L)(NO_3_)_2_] (L = 2,2′-(propane-1,2-diylbis((nitrilo)methylylidene))bis(6-(methoxy)phenolato)) have been reported.^72^ A group of Ni(II)-Pr(III) compounds consisting of 21 heterometallic clusters, which are mainly based on Schiff base,^73−76^ salen,^77−79^ and other phenolate ligands, has been reported.^80−82^ A large family of Mn(II/III)-Ln(III) heterometallic clusters dominated by heteroleptic Schiff, Mannich base-carboxylate, and nitrate derivatives has also been reported.^83−87^ Another group of Mn(II/III)-Ln(III) compounds consisting of various alkoxide-carboxylate-nitrate derivatives has been reported, i.e., [Ln_4_Mn_8_(μ_3_-O)_8_(hmp)_4_(benz)_12_(NO_3_)_4_(benzH)(EtOH)] (Ln = La, Pr, Nd, Gd, Dy; and benz = benzoate),^88^ [Ln_2_Mn_4_(μ_4_-O)_2_(H_2_edte)_2_(piv/benz)_8-*x*_(NO_3_)*_x_*] (H_2_edte = *N*,*N*,*N*′,*N*′-tetrakis(2-hydroxyethyl)ethylenediamine); (Ln = La, Ce, Pr, Nd, Eu, Sm, Gd, Tb, Dy, Ho, Er, Tm, Yb, and Y, and *x* = 0, 2).^89^ The presence of NO_3_^–^ or RCOO^–^ ligands in the lanthanide coordination sphere arose from the synthesis method applied, which involved the use of Mn(OOCR)_2_ and Ln(NO)_3_ as regents and amines to deprotonate the phenolic ligands. Therefore, **1**–**3** are uncommon examples of oxo-alkoxide clusters synthesized without using common methods. Furthermore, despite the compounds supported by 1,3-diketonato ligands, i.e., [Ln_2_Mn_2_(μ_3_-OMe)_2_(μ-OMe)_4_(dpm)_6_(HOMe)_*x*_] (Ln = Gd, Tb, Dy; *x* = 0, 2; dpm = 2,2,6,6-tetramethylheptane-3,5-dionato),^90^ [LnMn(μ-OMe)_2_(dpm)_4_(HOMe)_2_] (Ln = La, Pr, Eu),^91^ and [GdMn_3_(μ_4_-({OCH_2_CH_2_}_3_N))_2_(acac)_6_][Mn(acac)_3_],^92^ there are no known simple heterometallic alkoxides for the chosen elements.

**Figure 4:**
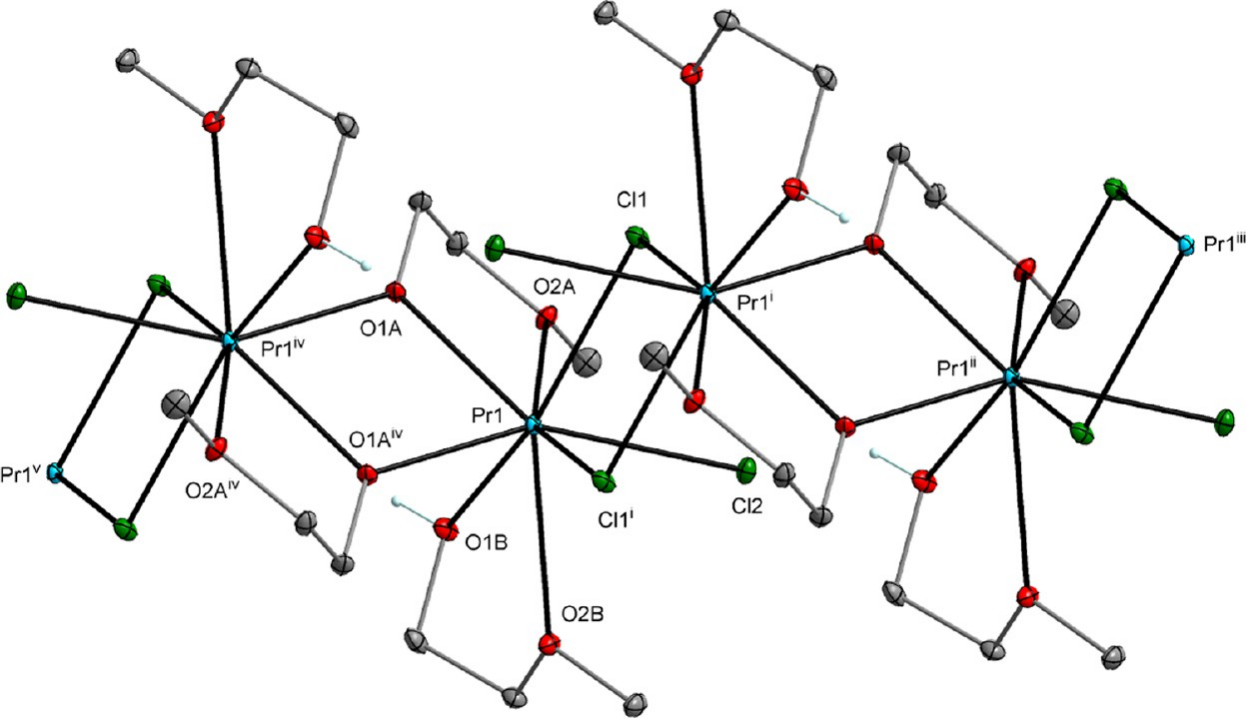
Molecular structure of [Pr(μ-OR)(μ-Cl)(HOR)Cl]*_n_* (**6**). Displacement ellipsoids are drawn at the 20% probability level. Hydrogen atoms of the alkyl groups are omitted for clarity [symmetry code: (i) −*x*+1, −*y*+1, −*z*+1, *z*; (ii) *x*+1, *y*, *z*; (iii) −*x*+2, −*y*+1, −*z*+1; (iv) −*x*, −*y*+1, −*z*+1; (v) *x*–1, *y*, *z*].

**Figure 5:**
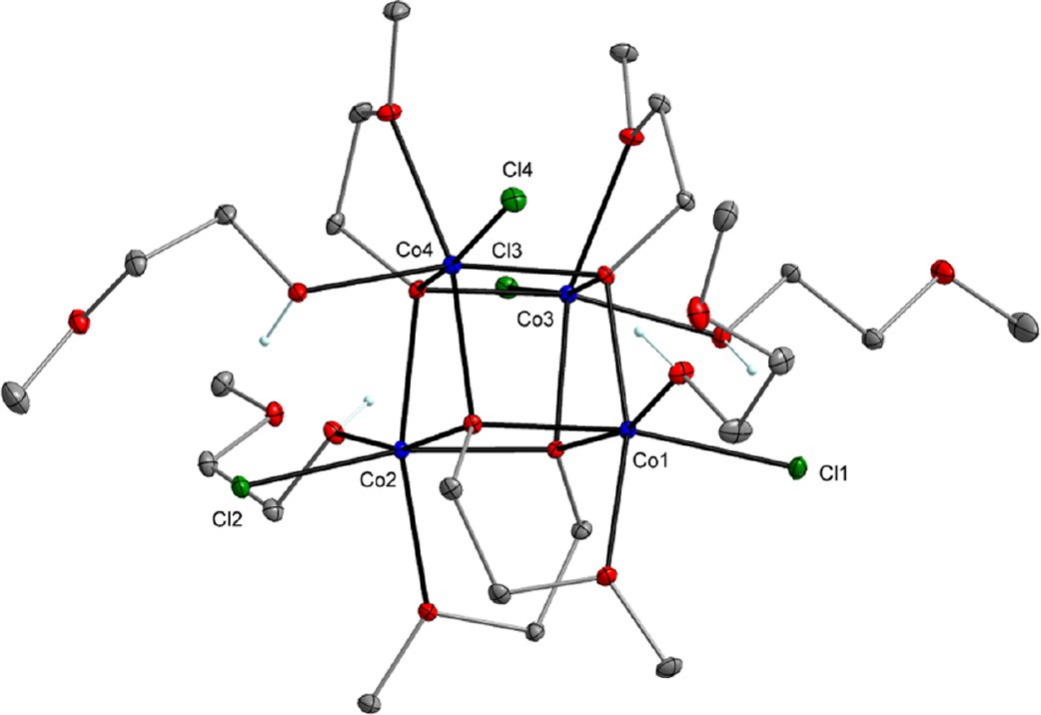
Molecular structure of [Co_4_(μ_3_-OR)_4_(HOR)_4_Cl_4_] (**7**). Displacement ellipsoids are drawn at the 20% probability level. Hydrogen atoms of the alkyl groups are omitted for clarity.

In studied reactions, the elimination of Cl^–^ from MCl_2_ and the release of H_2_ were observed, leading to the synthesis of homometallic chloro-alkoxides, which further interact in solution to form heterometallic lanthanide–transition-metal clusters. Strong evidence for this assumption was found when we partially reacted Pr with CoCl_2_, and besides the crystals of **4** (SI, Figure S1) the praseodymium(III) and cobalt(II) chloro-alkoxides of [Pr(μ-OR)(μ-Cl)(HOR)Cl]_n_ (**6**, 3%) and [Co_4_(μ_3_-OR)_4_(HOR)_4_Cl_4_] (**7**, 41%) were observed (Figures 4 and 5).

**Figure 6:**
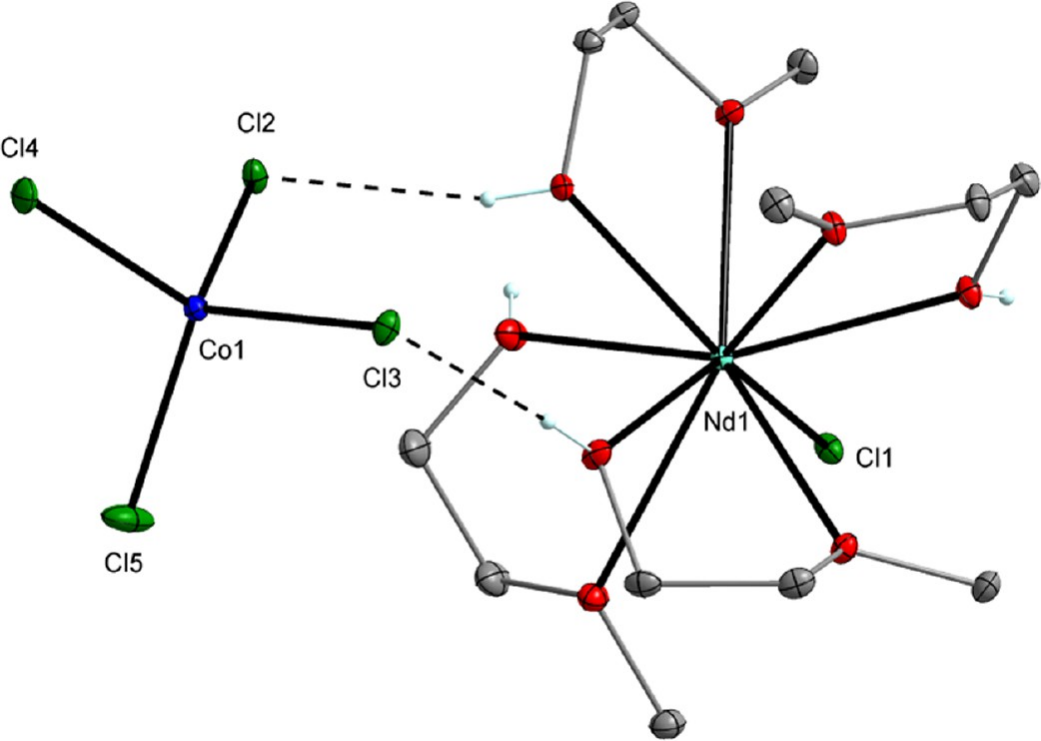
Molecular structure of [Nd(HOR)_4_Cl][CoCl_4_] (**10**). Displacement ellipsoids are drawn at the 30% probability level. Hydrogen atoms of the alkyl groups are omitted for clarity.

In a similar reaction performed in an ROH/EtOH mixture and using NiCl_2_ or MnCl_2_ instead of CoCl_2_, the formation of [Ni_4_(μ_3_-OR)_4_(HOEt)_4_Cl_4_] (**8**, 38%) or [Mn_4_(μ_3_-OR)_4_(HOR)_2_(HOEt)_2_Cl_4_] (**9**, 31%) were also detected (SI, Figures S4 and S5). The synthesis method developed shows that a metallic lanthanide is essential for forming homometallic intermediates, which further react to give molecular heterometallic clusters. The formation of M–OR or Ln–OR bonds and the migration of the Cl^–^ anion from M to Ln atoms prevent the synthesis of ionic byproducts. These can be easily formed by simple interaction between LnCl_3_ and MCl_2_. For example, the direct reaction between NdCl_3_ and CoCl_2_ can lead to the formation of the ionic compound [Nd(HOR)_4_Cl][CoCl_4_] (**10**, 82%) (Figure 6).

**Figure 7:**
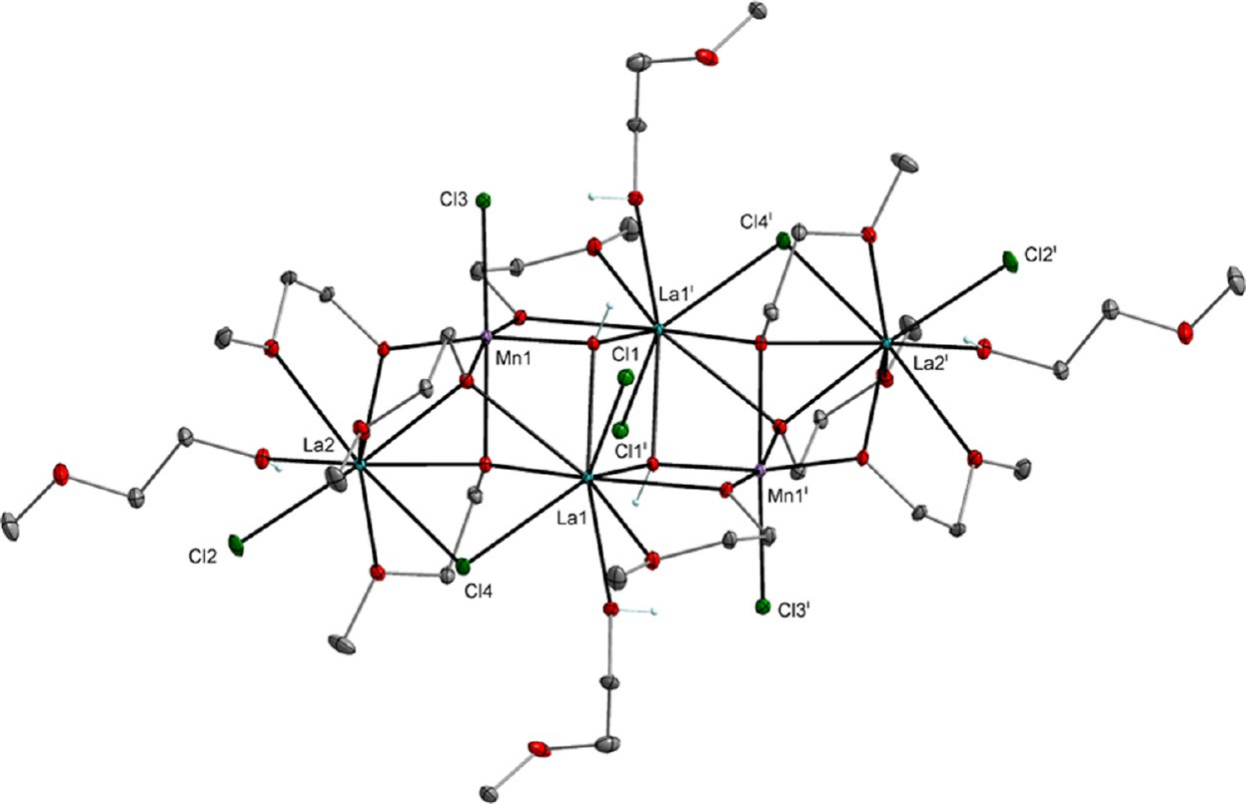
Molecular structure of [La_4_Mn_2_(μ_3_-OH)_2_(μ_3_-OR)_4_(μ-OR)_4_(μ-Cl)_2_(Cl)_6_(HOR)_4_] (**11**), which is isostructural with [Pr_4_Mn_2_(μ_3_-OH)_2_(μ_3_-OR)_4_(μ-OR)_4_(μ-Cl)_2_(Cl)_6_(HOR)_4_] (**12**) (SI, Figure S6). Displacement ellipsoids are drawn at the 30% probability level. Hydrogen atoms of the alkyl groups, solvent molecules, and the second part of the disordered lanthanum atoms and alkoxy ligands are omitted for clarity [symmetry code: (i) −*x*+1, −*y*+1, −*z*+1].

**Scheme 2:**
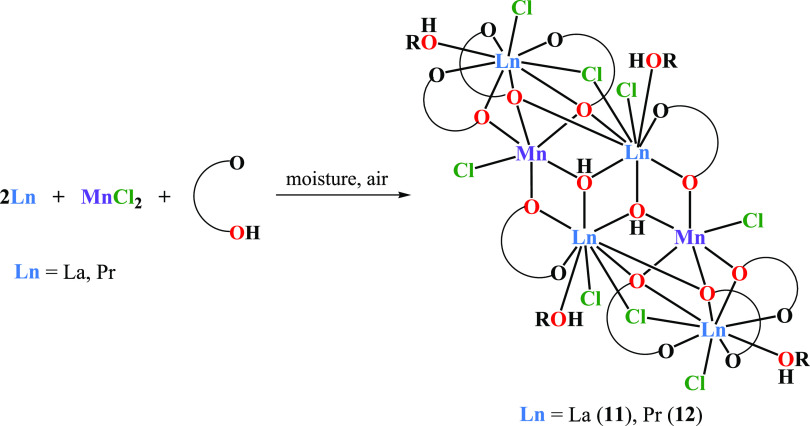
Synthesis of **11** and **12**

The heterometallic clusters [Ln_4_Mn_2_(μ_3_-OH)_2_(μ_3_-OR)_4_(μ-OR)_4_(μ-Cl)_2_(HOR)_4_Cl_6_] (Ln = La(**11**), 39%; and Pr(**12**), 35%) were formed when we neglected the strict anhydrous conditions and the reaction mixtures remained in contact with moisture and oxygen (Scheme 2, Figure 7).

Compounds **11** and **12** retain the stoichiometry of elements observed for **1**–**5**, i.e., Ln:M = 2:1, but the formation of μ_3_-OH bridges and Mn(III) centers are observed upon reaction with H_2_O and O_2_ (Figures 7 and S6). Structural analysis of the central motif observed in **11**–**12** suggests that it is formed by the interaction of the [Ln(OR)(HOR)Cl_2_] species observed in **6** with the double-open dicubane unit [Ln_2_Mn_2_(OH)_2_(OR)_4_(HOR)_2_Cl_4_], leading to the formation of two μ_3_-OR and one μ-Cl bridge. Ln(III) ions coordinated by seven oxygen and two chlorine atoms adopt capped square antiprismatic or muffin geometries (SI, Table S2). Analytical methods and infrared (IR) spectroscopy were used to characterize **1**–**12** (SI, Figures S7–S18).

### Synthesis of Oxide Materials by Thermal Decomposition of **1–5** and **12**, as well as Powder X-ray Diffraction and Transmission Electron Microscopy Study of the Resulting Materials

**Figure 8:**
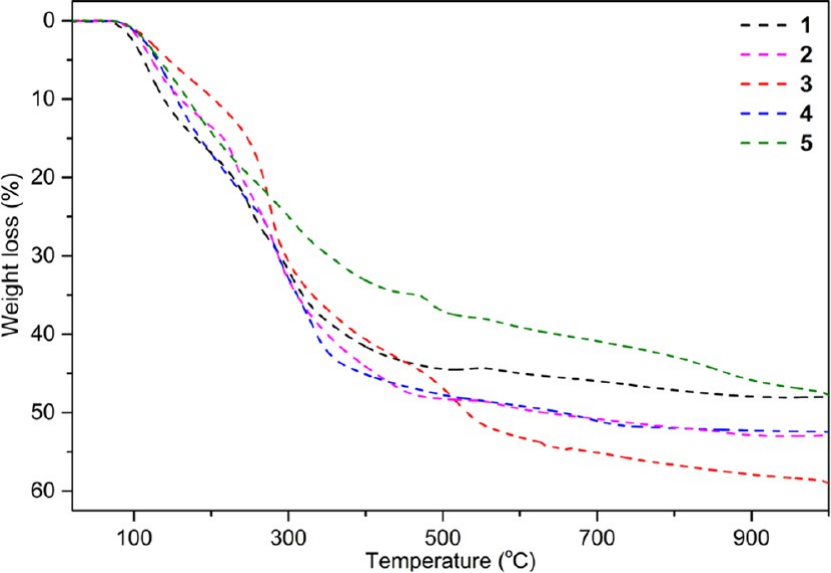
Thermogravimetric analysis (TGA) curves (dashed lines) for **1**–**5** recorded at a heating rate of 10 °C min^–1^ in argon/air atmosphere over the temperature range 20–1000 °C.

A combination of thermogravimetry, differential thermal analysis (TG-DTA), and powder X-ray diffraction (PXRD) was used to study the thermal decomposition of **1**–**5** to identify appropriate temperatures for removing organic ligands and to investigate the metal oxide phases formed. The decomposition behavior of the resulting compounds from 20 to 1000 °C at a heating rate of 10 °C min^–1^ under an argon/air atmosphere was investigated. Representative thermograms for the isostructural compounds **1–5** are shown in Figure 8.

The results indicate that the heterometallic clusters undergo multistep decomposition. All of the compounds are thermally stable up to 90 °C. Above this temperature, the alkoxide ligands start to be eliminated from the metal coordination sphere. The organic ligand decomposition products were efficiently removed up to 360 °C, leading to mixtures of LnOCl and MO oxides. The presence of LnOCl and MO/M_2_O_3_ or MO·M_2_O_3_ oxide phases in the range 500–700 °C was additionally confirmed by PXRD analysis of the solid residues obtained when the thermal decomposition of **1–5** was stopped at 500–600 °C. Above 750 °C, the powder diffractograms reveal the presence of heterometallic oxide phases. The total mass losses of 47.6% for **1**, 52.9% for **2**, 58.7% for **3**, and 52.4% for **4**, correspond well with the estimated values of 48.1, 53.1, 59.8, and 52.9% for a mixture of LnOCl and LnMO_3_. For **5**, the total mass losses of 47.5% suggest the formation of a more complex mixture of products.

**Figure 9:**
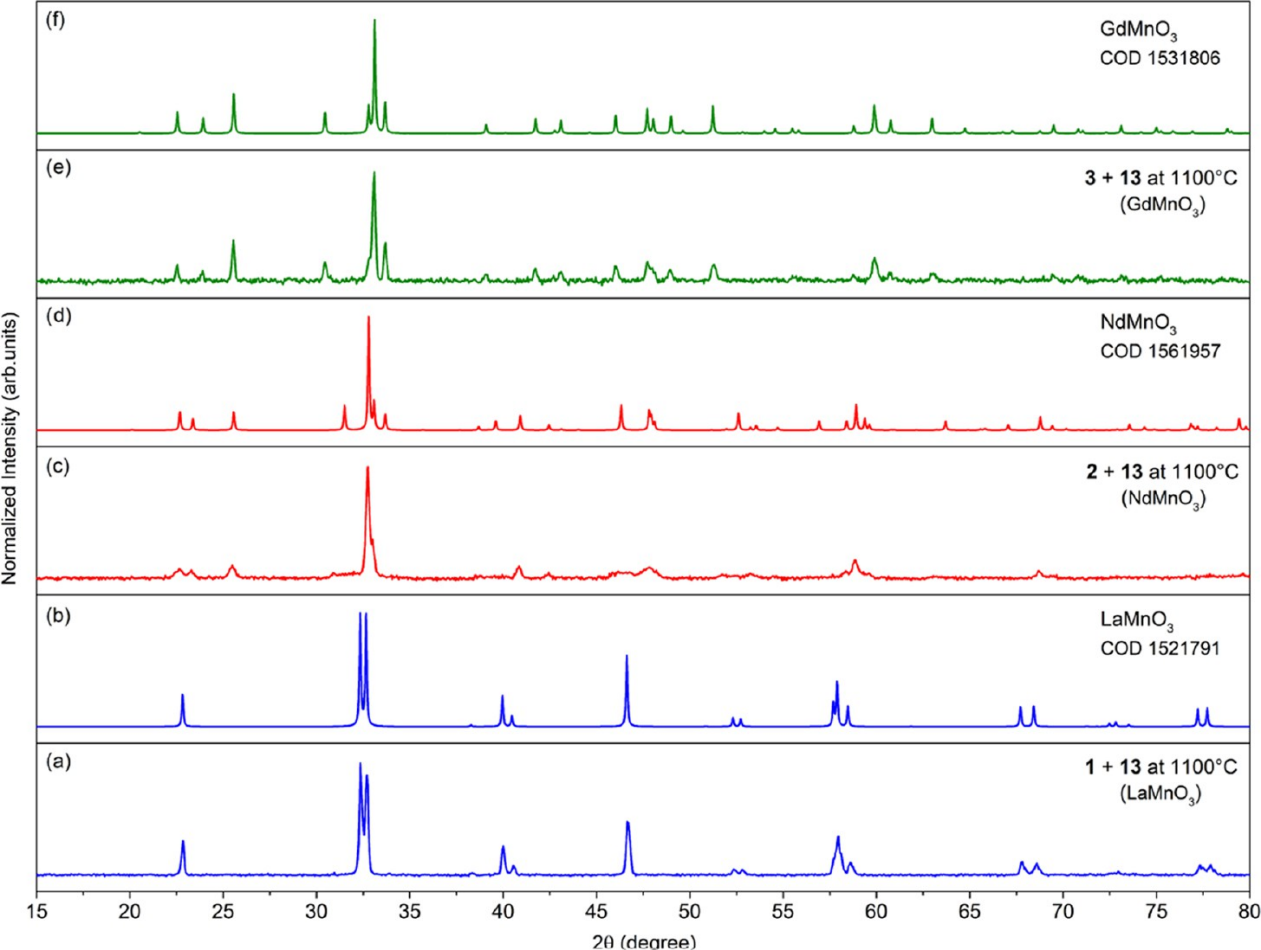
PXRD patterns of oxide materials prepared by calcination of **1–3** and **13** at 1100 °C (a, c, e), LaMnO_3_ [COD 2022; 1521791] (b), NdMnO_3_ [1561957] (d), GdMnO_3_ [1531806] (f).

**Figure 10:**
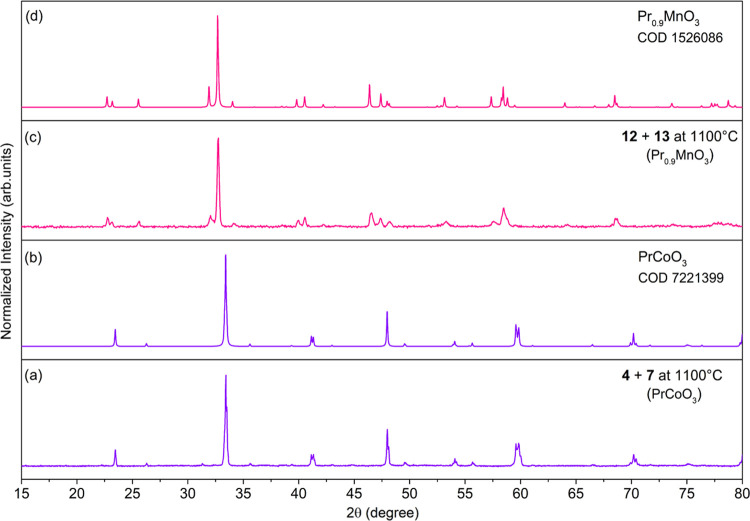
PXRD patterns of oxide materials prepared by calcination at 1100 °C of a mixture of **4** and **7** (a) or **12** and **13** (c), PrCoO_3_ [COD 2021; 7221399] (b), and Pr_0.9_MnO_3_ [1526086] (d).

The PXRD patterns of the oxide materials synthesized by calcination of **1–3** and **12** at 1100 °C show the formation of mixtures of LnMnO_3_ and LnOCl or Ln_2_O_3_ (SI, Figures S19–S22). Within the series of resulting oxides, the heterometallic perovskite-type LnMnO_3_ species are promising for a wide range of practical applications as biosensors for food safety and environmental monitoring,^93^ cathode materials for Zn–air batteries,^94^ and multiferroic materials.^95,96^ The metal cation stoichiometry Ln:M = 2:1 in the molecular precursors of **1–3** and **12** cause the formed LnMnO_3_ phases to be contaminated by the LnOCl, Ln_2_O_3_, or LnO_2_ (SI, Figures S19–S22). After analysis of the above results, we decided to use [MnCl_2_(HOR)]*_n_* (**13**) as an additional source of Mn(II) ions to decrease the Ln(III) excess during the synthesis of mixed-metal materials (SI, Figures S23 and S24). The selective formation of heterometallic phase peaks is detected when **13** is sintered together with **1–3** at the reactant stoichiometry Ln:Mn = 1:1. LaMnO_3_ crystallizes as a trigonal perovskite structure, while GdMnO_3_ and NdMnO_3_ are orthorhombic (Figures 9 and 10). For the combination of **12** and **13**, the formation of mixed-valence Mn(III)–Mn(IV) praseodymium manganite with composition Pr_0.9_MnO_3_ was observed.

A mixture of PrOCl, PrO_2_, and PrCoO_3_ is formed during the thermal decomposition of **4** (SI, Figure S25). When calcination of **4** is performed together with **7** as an external Co(II) source, the crystallization of PrCoO_3_ is observed (Figure 10). Mixed-metal oxides of Pr(III) and Co(III) have been investigated as catalysts for the oxidation of ethane or CO,^97,98^ and as ferroelectric and magnetic materials.^99^

**Figure 11:**
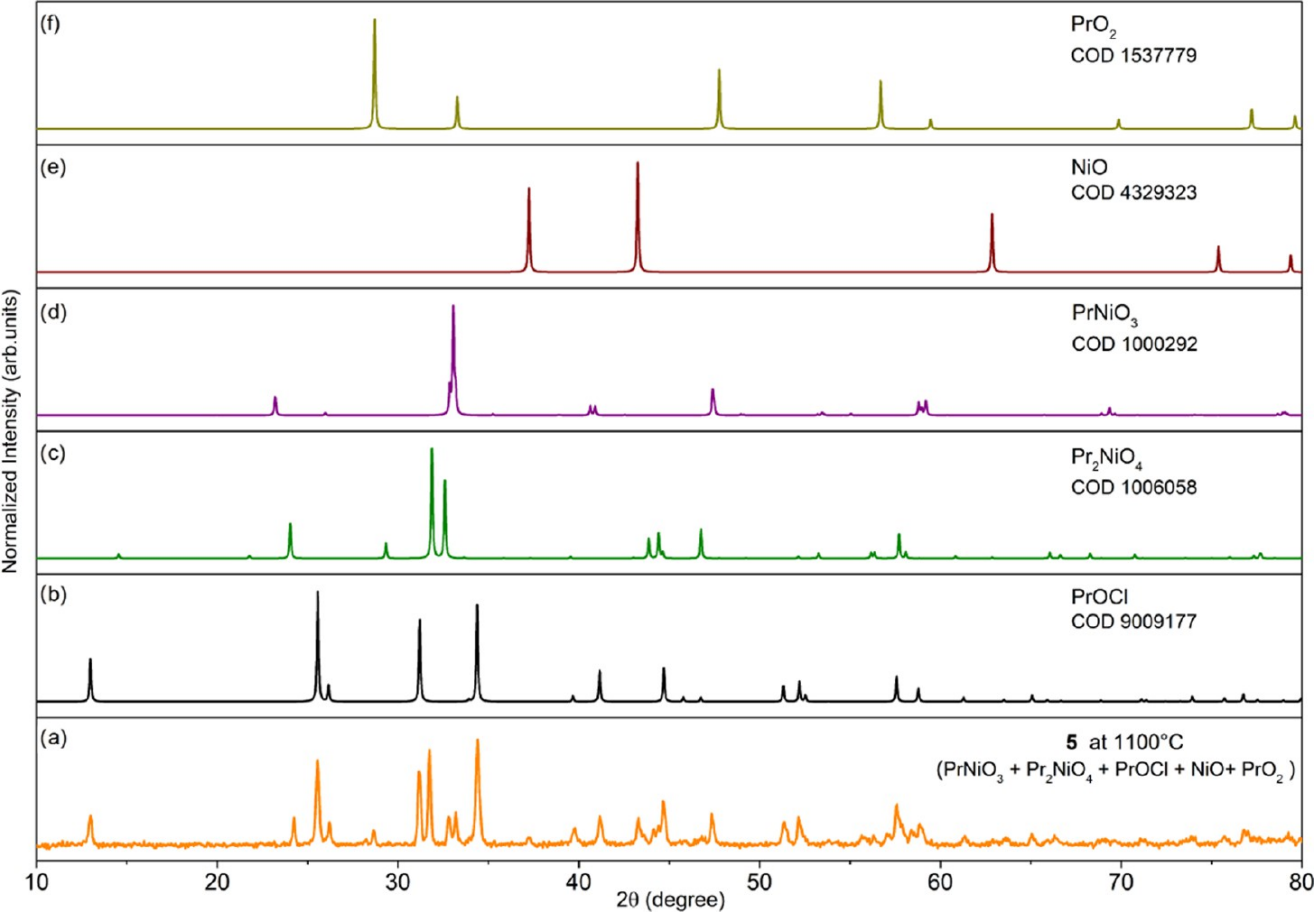
PXRD patterns of oxide materials prepared by calcination of **5** at 1100 °C (a), PrOCl [9009177] (b), Pr_2_NiO_4_ [1006058] (c), PrNiO_3_ [1000292] (d), NiO [4329323] (e), PrO_2_ [1537779] (f).

Compound **5** decomposes into a mixture of the homo- and heterometallic oxides PrOCl, PrO_2_, NiO, PrNiO_3_, and Pr_2_NiO_4_ with the metal cations in different oxidation states, i.e., Pr(III/IV) and Ni(II/III) (Figure 11). When we performed the thermolysis of **5** using [NiCl_2_(HOR)_2_] (**14**; SI, Figures S26 and S27) as additional Ni(II) sources, the formation of a mixture of the same products was observed (SI, Figure S28).

**Figure 12:**
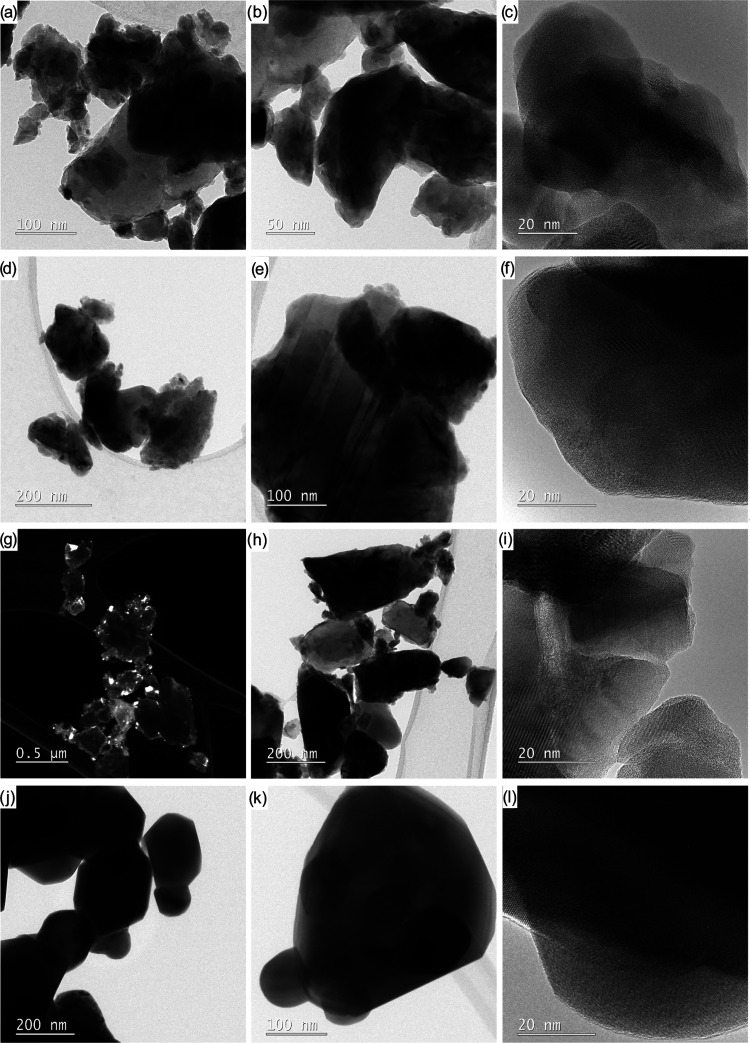
TEM images of oxide materials prepared by calcination of **13** with **1** (LaMnO_3_, a–c), **2** (NdMnO_3_, d–f), **3** (GdMnO_3_, g–i), or **12** (Pr_0.9_MnO_3_, j–l) at 1100 °C.

**Figure 13:**
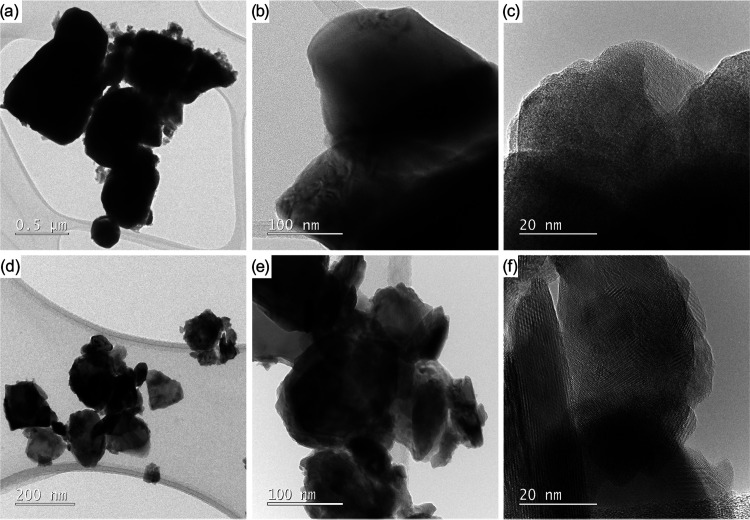
TEM images of oxide materials prepared by calcination of **4** and **7** (PrCoO_3_, a–c), or **5** (a mixture of PrOCl, Pr_2_NiO_4_, PrNiO_3_, NiO, and PrO_2_; d–f) at 1100 °C.

Transmission electron microscopy (TEM) and high-resolution (HR)TEM were employed to study the shapes, sizes, and lattice arrangements of the synthesized nanoparticles (Figures 12 and 13). TEM analysis of the sample annealed at 1100 °C clearly shows that it comprises agglomerated oval nanoparticles with diameters in the range 25–190 nm for LaMnO_3_, 63–158 nm for NdMnO_3_, 10–101 nm for GdMnO_3_, 31–201 nm for Pr_0.9_MnO_3_, and 64–216 nm for PrCoO_3_. For LaMnO_3_, the crystal lattices with d-spacings of 0.389 and 0.277 nm belong to the (21̅0) and (102̅) crystal planes of rhombohedral perovskite (Figure 12a–c).

The orthorhombic perovskite structures were confirmed by the determination of space distances characteristic of the (101) and (210) planes, which are 0.395 and 0.273 nm for NdMnO_3_, 0.394 and 0.273 for GdMnO_3_, and 0.391 and 0.263 nm for Pr_0.9_MnO_3_ (Figure 12d–l), respectively. For orthorhombic PrCoO_3_, the measured lattice fringe spacings of 0.379 and 0.268 nm are consistent with the d values for the (110) and (112) planes (Figure 13a–c).

The elemental compositions of the resulting nanomaterials were investigated by energy-dispersive spectrometry (EDS) and inductively coupled plasma (ICP) analysis, revealing the formation of LaMnO_3_, NdMnO_3_, GdMnO_3_, and PrCoO_3_ perovskites with an Ln:M ratio of 1:1 (SI, Figures S29–S32). EDS and ICP measurements also confirmed the formation of praseodymium deficient Pr_0.9_MnO_3_ containing ∼30% of Mn(IV) (SI, Figure S33). EDS analysis of the oxide material derived from the decomposition of **5** confirms the presence of particles with different Pr:Ni contents, which agrees well with the mixture of homo- and heterometallic phases observed by PXRD study (PrOCl, NiO, PrO_2_, Pr_2_NiO_4_, and PrNiO_3_; SI, Figures S34–S36).

## Conclusions

We have developed a simple and efficient synthetic strategy for the preparation of the heterometallic 3d–4f alkoxides [Ln_4_Mn_2_(μ_6_-O)(μ_3_-OR)_8_(HOR)_*x*_Cl_6_] (Ln = La (**1**), Nd (**2**), Gd (**3**); *x* = 0, 2, 4); [Pr_4_M_2_(μ_6_-O)(μ_3_-OR)_8_(HOR)*_x_*Cl_6_] (M = Co (**4**), Ni (**5**), and *x* = 2, 4); and [Pr_4_Mn_2_(μ_3_-OH)_2_(μ_3_-OR)_4_(μ-OR)_4_(μ-Cl)_2_(HOR)_4_Cl_6_] (**12**) by the direct reaction of metallic lanthanides (La, Pr, Nd, Gd) with divalent transition-metal chlorides (MCl_2_, where M(II) = Mn(II), Ni(II), or Co(II)) using 2-methoxyethanol (ROH) as the solvent and ligand precursor. This method is an attractive alternative to commonly used methods involving the direct reaction of Mn(OOCR)_2_, Ln(NO)_3_, phenol, or alcohol derivative ligands, and with amines as deprotonation agents. We showed a new and controlled way for rationally designing 3d–4f clusters based on the hexanuclear Ln_4_M_2_(μ_6_-O) motif. One of the main challenges in synthesizing heterometallic 3d–4f compounds is the different chemical properties, coordination capabilities, and ionic radii of the 3d and 4f metal ions. In the field of coordination chemistry, **4** and **5** are the first reported alkoxides of Co(II)/Ni(II) and Pr(III).

The obtained heterometallic clusters **1**–**5** and **12** were used as molecular platforms to investigate the solid-state phase compositions, homogeneities, and morphologies of the synthesized oxide materials. Thermal decomposition of **1–4** and **12** led to the formation of mixtures of LnMO_3_ and LnOCl or Ln_2_O_3_. The calcination of **1–4** and **12** at 1100 °C in the presence of an external M(II) source led to the selective formation of industrially important heterometallic perovskite-type materials of LaMnO_3_, GdMnO_3_, NdMnO_3_, Pr_0.9_MnO_3_, and PrCoO_3_. Compound **5** decomposed into a mixture of homo- and heterometallic oxides including PrOCl, PrO_2_, NiO, PrNiO_3_, and Pr_2_NiO_4_ with the metal cations Pr(III/IV) and Ni(II/III) adopting different oxidation states.

The resulting transition-metal–lanthanide clusters are interesting molecular materials with potential applications in photocatalysis, photoluminescence, and electronic devices. We have also established that they may also act as efficient molecular precursors for the preparation of advanced multicomponent oxide materials with a large variety of chemical and physical properties, which will be essential for further applications.

## Experimental Section

All syntheses were performed with Schlenk techniques under a dry nitrogen atmosphere. All reagents were obtained from commercial sources: 2-methoxyethanol, ethanol, methanol, tetrahydrofuran, hexane, toluene, MnCl_2_, CoCl_2_, and NiCl_2_ were obtained from Sigma-Aldrich and the metallic elements were obtained from Alfa Aesar. Solvents were purified using standard methods and distilled under a dry nitrogen atmosphere before use. Hexane and toluene were purified by refluxing over sodium, tetrahydrofuran (THF) was distilled from sodium/benzophenone, while methanol and ethanol were refluxed and distilled over metallic magnesium. Fourier transform infrared attenuated total reflectance (FTIR-ATR) spectra were recorded on a Bruker Vertex 70 Vacuum spectrometer. Elemental analysis was performed with a PerkinElmer 2400 CHN elemental analyzer. Metal ion concentrations were determined by inductively coupled plasma optical emission spectrometry using an ICP-OES −iCAP 7400 Duo spectrometer (Thermo Scientific). Chlorine analysis was performed by the Schöninger method using an Elemental Micro K analyzer. TG-DTA measurements were carried out using a Setaram 96 system under an argon atmosphere at a heating rate of 10 °C min^–1^. The thermal decomposition of metal alkoxide precursors was performed using an NT 1313 furnace (Neotherm) equipped with a KXP4 thermostat under atmospheric air. The resulting powders were investigated by powder XRD analysis using a Philips X’PERT diffractometer. The sample was analyzed using the crystallography open database (COD). The morphologies of the resulting oxide materials were examined using an FEI Tecnai G^2^ 20 X-Twin TEM microscope equipped with a field emission gun (FEG) and an integrated energy-dispersive spectrometer (EDAX), or FEI Titan G2 60-300 HR-TEM microscope with spectrometer electron energy-loss spectroscopy (EELS). For the TEM observation, 200-mesh copper grids with lacey carbon films were used. The signals from copper and carbon in the grid can be visible in TEM-energy-dispersive X-ray spectroscopy (EDX) spectra. Single-crystal XRD analyses were performed using a PX Xcalibur (for **1**, **11** at 100 K), Xcalibur Ruby (for **2** at 80 K, **3** at 90 K, **4**, **4a**, **5**, **10**, **13** at 100 K; **7** at 240 K), XtaLAB Synergy R (for **6**, **8**, **9**, **12** at 100 K) and Kuma KM4 (for **14** at 100 K) diffractometers.^100^ Crystal data, experimental data collection, and structure refinement details are summarized in Table S1. The structures were solved by direct methods and refined by the full-matrix least-squares on *F*^2^ using the SHELXTL package.^101^ Nonhydrogen atoms were located in successive difference Fourier syntheses and refined with anisotropic thermal parameters on *F*^2^. All hydrogen atoms were generated theoretically and added to the structure factor calculations but not refined. Molecular graphics for the resulting structures were created using Diamond (version 3.1e).^102^

### Synthesis of [La_4_Mn_2_(μ_6_-O)(μ_3_-OR)_8_(HOR)_4_Cl_6_] (**1**)

A 150 mL Schlenk flask equipped with a stir bar was charged with metallic lanthanum (0.93 g; 6.65 mmol), 2-methoxyethanol (10 mL), methanol (10 mL), and MnCl_2_ (0.42 g; 3.33 mmol). For the activation of metallic lanthanum, two crystals of I_2_ were used. The mixture was vigorously stirred at room temperature within 54 h and then filtered off. The resulting dark brown filtrate was concentrated under a vacuum and left at room temperature for crystallization. Colorless crystals of **1** were grown at ambient temperature within a few weeks (3–4). The crystals were filtered off, washed with hexane (3 × 5 mL), and dried under vacuum. Overall yield: 1.39 g (0.77 mmol, 46% calculated using MnCl_2_ as limiting reagent); Anal. Calcd for C_36_H_88_O_25_Cl_6_Mn_2_La_4_: C, 24.03; H, 4.93; Cl, 11.82; Mn, 6.11; La, 30.88. Found: C, 24.11; H, 4.94; Cl, 11.79; Mn, 6.14; La, 30.96. FTIR-ATR (cm^–1^): 3397 (w), 2923 (m), 2866 (m), 2836 (m), 2706 (vw), 1589 (vw), 1453 (w), 1390 (w), 1361 (w), 1242 (w), 1195 (w), 1100 (m), 1048 (vs), 1012 (s), 903 (m), 831 (m), 568 (m), 511 (m), 420 (w).

### Synthesis of [Nd_4_Mn_2_(μ_6_-O)(μ_3_-OR)_8_(HOR)_2_Cl_6_] (**2**)

A 150 mL Schlenk flask equipped with a stir bar was charged with metallic neodymium (1.19 g; 8.3 mmol), 2-methoxyethanol (15 mL), methanol (10 mL), and MnCl_2_ (0.53 g; 4.2 mmol). For the activation of metallic neodymium, two crystals of I_2_ were used. The mixture was stirred at room temperature within 76 h and then filtered off. The resulting dark brown filtrate was concentrated under a vacuum and left at room temperature for crystallization. Light-pink crystals of **2** were grown at ambient temperature within 8 weeks. The crystals were filtered off, washed with hexane (3 × 5 mL), and dried under vacuum. Overall yield: 1.78 g (1.07 mmol, 51% calculated using MnCl_2_ as limiting reagent); Anal. Calcd for C_30_H_72_O_21_Cl_6_Mn_2_Nd_4_: C, 21.60; H, 4.35; Cl, 12.75; Mn, 6.59; Nd, 34.58. Found: C, 21.57; H, 4.33; Cl, 12.70; Mn, 6.54; Nd, 34.52. FTIR-ATR (cm^–1^): 3340 (m), 3206 (m), 2961 (w), 2941 (w), 2900 (w), 2837 (m), 1596 (m), 1443 (m), 1422 (w), 1343 (w), 1318 (w), 1259 (m), 1196 (w), 1085 (s), 1041 (vs), 978 (m), 888 (w), 794 (m), 684 (w), 661 (w), 589 (w), 519 (w).

### Synthesis of [Gd_4_Mn_2_(μ_6_-O)(μ_3_-OR)_8_Cl_6_] (**3**)

A 150 mL Schlenk flask equipped with a stir bar was charged with metallic gadolinium (1.34 g; 8.5 mmol), 2-methoxyethanol (12 mL), methanol (5 mL), and MnCl_2_ (0.54 g; 4.3 mmol). The mixture was vigorously stirred at room temperature within 72 h and then filtered off. The resulting dark yellow filtrate was concentrated under a vacuum and left at room temperature. Colorless crystals of **3** were grown at ambient temperature within 3 weeks. The crystals were filtered off, washed with hexane (3 × 5 mL), and dried under vacuum. Overall yield: 1.54 g (0.98 mmol, 46% calculated using MnCl_2_ as limiting reagent); Anal. Calcd for C_24_H_56_O_17_Cl_6_Mn_2_Gd_4_: C, 18.38; H, 3.60; Cl, 13.56; Mn, 7.01; Gd, 40.11. Found: C, 18.44; H, 3.61; Cl, 13.49; Mn, 6.96; Gd, 40.18. FTIR-ATR (cm^–1^): 3412 (vw), 3076 (vw), 3025 (vw), 2927 (w), 2865 (w), 2837 (w), 2705 (vw), 1592 (vw), 1454 (w), 1391 (vw), 1359 (vw), 1264 (w), 1239 (w), 1196 (vw), 1159 (vw), 1094 (m), 1039 (vs), 1009 (s), 913 (m), 830 (m), 621 (w), 581 (m), 462 (m), 418 (w).

### Synthesis of [Pr_4_Co_2_(μ_6_-O)(μ_3_-OR)_8_(HOR)_2_Cl_6_] (**4**)

A 150 mL Schlenk flask equipped with a stir bar was charged with metallic praseodymium (1.12 g; 8.0 mmol), 2-methoxyethanol (12 mL), methanol (10 mL), and CoCl_2_ (0.52 g; 4.0 mmol). The mixture was stirred at room temperature within 72 h. Then, the mixture was filtered off, and the resulting dark blue solution was concentrated under a vacuum and left at room temperature. Blue crystals of **4** were grown at ambient temperature within 6 weeks. The crystals were filtered off, washed with hexane (3 × 5 mL), and dried under vacuum. Overall yield: 1.55 g (0.93 mmol, 47% calculated using CoCl_2_ as limiting reagent); Anal. Calcd for C_30_H_72_O_21_Cl_6_Co_2_Pr_4_: C, 21.67; H, 4.36; Cl, 12.79; Co, 7.09; Pr, 33.89. Found: C, 21.63; H, 4.35; Cl, 12.73; Co, 7.11; Pr, 33.93. FTIR-ATR (cm^–1^): 3433 (w), 2866 (m), 2834 (m), 2703 (vw), 1592 (w), 1454 (w), 1390 (w), 1361 (w), 1270 (vw), 1242 (w), 1196 (w), 1097 (m), 1046 (vs), 1011 (s), 906 (m), 830 (m), 570 (m), 470 (vw), 420 (w).

### Synthesis of [Pr_4_Ni_2_(μ_6_-O)(μ_3_-OR)_8_(HOR)_4_Cl_6_] (**5**)

A 150 mL Schlenk flask equipped with a stir bar was charged with metallic praseodymium (1.06 g; 7.5 mmol), 2-methoxyethanol (12 mL), methanol (5 mL), and NiCl_2_ (0.49 g; 3.8 mmol). The mixture was stirred at room temperature within 72 h. Then, the mixture was filtered off, and the resulting light green solution was concentrated under vacuum to half the volume and left at room temperature. Green crystals of **5** were grown at ambient temperature within 8 weeks. The crystals were filtered off, washed with hexane (3 × 5 mL), and dried under vacuum. Overall yield: 1.51 g (0.83 mmol, 44% calculated using NiCl_2_ as limiting reagent); Anal. Calcd for C_36_H_88_O_25_Cl_6_Ni_2_Pr_4_: C, 23.83; H, 4.89; Cl, 11.72; Ni, 6.47; Pr, 31.06. Found: C, 23.78; H, 4.88; Cl, 11.69; Ni, 6.44; Pr, 31.10. FTIR-ATR (cm^–1^): 3403 (w), 2815 (m), 2697 (w), 1607 (vw), 1452 (w), 1383 (w), 1359 (w), 1322 (vw), 1271 (vw), 1239 (w), 1195 (w), 1105 (s), 1058 (vs), 1012 (s), 962 (w), 907 (m), 832 (m), 689 (vw), 600 (vw), 561 (m), 515 (w), 474 (vw), 443 (w).

### Synthesis of [Pr(μ-OR)(μ-Cl)(HOR)Cl]*_n_* (**6**) and [Co_4_(μ_3_-OR)_4_(HOR)_4_Cl_4_] (**7**)

The reaction was performed under identical conditions as described for the synthesis of compound **4**. However, after 4 h, the excess unreacted metallic praseodymium was removed from the reaction vessel, and the reaction mixture was filtered off and allowed to crystallize under ambient conditions. After a few weeks, a mixture of the three types of crystals was obtained. In addition to the previously described compound **4**, colorless crystals of compound **6** and blue crystals of compound **7** were obtained. Compounds **6** and **7** for spectroscopic studies were isolated from the reaction mixture, but their yields were not determined.

### [Pr(μ-OR)(μ-Cl)(HOR)Cl]*_n_* (**6**)

Overall yield: 0.10 g (0.28 mmol, 7% calculated using CoCl_2_ as limiting reagent); Anal. Calcd for (C_6_H_15_O_4_Cl_2_Pr)*_n_*: C, 19.85; H, 4.17; Cl, 19.53. Found: C, 19.81; H, 4.16; Cl, 19.48. FTIR-ATR (cm^–1^): 3254 (m), 2926 (w), 2906 (w), 2834 (w), 1648 (w), 1454 (w), 1371 (w), 1316 (w), 1275 (vw), 1240 (w), 1194 (w), 1162 (vw), 1101 (m), 1086 (m), 1051 (vs), 1011 (m), 976 (w), 914 (m), 904 (m), 835 (m), 574 (m), 551 (m), 439 (m).

### [Co_4_(μ_3_-OR)_4_(HOR)_4_Cl_4_] (**7**)

Overall yield: 0.41 g (0.42 mmol, 41% calculated using CoCl_2_ as limiting reagent); Anal. Calcd for C_24_H_60_O_16_Cl_4_Co_4_: C, 29.35; H, 6.16; Cl, 14.44. Found: C, 29.30; H, 6.14; Cl, 14.48. FTIR-ATR (cm^–1^): 3552 (m), 3360 (w), 2934 (w), 2881 (w), 2831 (w), 1642 (w), 1454 (w), 1369 (w), 1232 (w), 1193 (w), 1115 (m), 1056 (vs), 1013 (m), 975 (w), 890 (w), 831 (m), 723 (m) 541 (w), 412 (m).

### Synthesis of [Pr(μ-OR)(μ-Cl)(HOR)Cl]*_n_* (**6**) and [Ni_4_(μ_3_-OR)_4_(HOEt)_4_Cl_4_] (**8**) or [Mn_4_(μ_3_-OR)_4_(HOR)_2_(HOEt)_2_Cl_4_] (**9**)

The reactions were performed under identical conditions as described for the synthesis of the mixture of compounds **4**, **6**, and **7**. A 150 mL Schlenk flask equipped with a stir bar was charged with metallic praseodymium (1.12 g; 8.0 mmol), 2-methoxyethanol (12 mL), ethanol (10 mL), and NiCl_2_ (0.52 g; 4.0 mmol) or MnCl_2_ (0.50 g; 4.0 mmol). The mixtures were stirred at room temperature within 7–8 h. Then, the excess unreacted metallic praseodymium was removed from the reaction vessel, and the reaction mixture was filtered off and allowed to crystallize under ambient conditions. After a few weeks, compounds **8** and **9** were isolated from the reaction mixtures.

### [Ni_4_(μ_3_-OR)_4_(HOEt)_4_Cl_4_] (**8**)

Overall yield: 0.31 g (0.38 mmol, 38% calculated using NiCl_2_ as limiting reagent); Anal. Calcd for C_20_H_52_O_12_Cl_4_Ni_4_: C, 27.89; H, 6.09; Cl, 16.47. Found: C, 27.82; H, 6.07; Cl, 16.43. FTIR-ATR (cm^–1^): 3239 (m), 2938 (w), 2877 (w), 2830 (w), 2714 (vw), 1613 (vw), 1448 (w), 1402 (w), 1364 (w), 1263 (w), 1249 (w), 1201 (w), 1163 (vw), 1120 (m), 1101 (m), 1051 (vs), 1011 (m), 909 (m), 884 (w), 836 (m), 661 (w), 593 (m), 459 (m), 407 (m).

### [Mn_4_(μ_3_-OR)_4_(HOR)_2_(HOEt)_2_Cl_4_] (**9**)

Overall yield: 0.28 g (0.31 mmol, 31% calculated using MnCl_2_ as limiting reagent); Anal. Calcd for C_22_H_56_O_14_Cl_4_Mn_4_: C, 29.16; H, 6.23; Cl, 15.65. Found: C, 29.07; H, 6.21; Cl, 15.60. FTIR-ATR (cm^–1^): 3507 (m), 3177 (w), 3153 (w), 3074 (vw), 2930 (w), 2889 (w), 2822 (w), 1727 (w), 1672 (m), 1614 (m), 1585 (w), 1546 (vw), 1485 (m), 1454 (m), 1410 (w), 1375 (m), 1352 (vw), 1324 (m), 1298 (s), 1248 (m), 1213 (m), 1157 (m), 1125 (m), 1086 (s), 1030 (m), 984 (w), 954 (vw), 892 (w), 865 (w), 838 (w), 790 (w), 755 (m), 740 (s), 699 (vs), 666 (m), 627 (vw), 565 (w), 530 (m), 507 (w), 442 (vw), 410 (m).

### Synthesis of [Nd(HOR)_4_Cl][CoCl_4_] (**10**)

A 150 mL Schlenk flask equipped with a stir bar was charged with NdCl_3_ (0.5 g; 2.0 mmol), 2-methoxyethanol (5 mL), and CoCl_2_ (0.26 g; 2.0 mmol). The mixture was stirred at room temperature within 8 h. The resulting violet solution was concentrated under a vacuum and left at room temperature. Green crystals of **10** were grown at ambient temperature within 2 weeks. The crystals were filtered off, washed with hexane (3 × 5 mL), and dried under vacuum. Overall yield: 1.12 g (1.60 mmol, 82%); Anal. Calcd for C_12_H_32_O_8_Cl_5_CoNd: C, 21.05; H, 4.71; Cl, 25.88; Co, 8.61; Nd, 21.06. Found: C, 21.09; H, 4.71; Cl, 25.76; Co, 8.68; Nd, 21.17. FTIR-ATR (cm^–1^): 3348 (m), 3255 (m), 3243 (m), 3231 (m), 3196 (m), 3008 (vw), 2958 (w), 2946 (w), 2892 (w), 2844 (w), 2796 (vw), 2746 (vw), 2646 (vw), 2604 (vw), 2490 (vw), 1625 (w), 1456 (m), 1402 (w), 1371 (w), 1326 (w), 1297 (w), 1275 (vw), 1262 (vw), 1229 (w), 1201 (w), 1184 (w), 1112 (w), 1081 (m), 1027 (vs), 998 (s), 974 (m), 900 (m), 819 (m), 566 (m), 550 (m), 524 (m), 421 (vw).

### Synthesis of [La_4_Mn_2_(μ_3_-OH)_2_(μ_3_-OR)_4_(μ-OR)_4_(μ-Cl)_2_(HOR)_4_Cl_6_] (**11**)

A 150 mL Schlenk flask equipped with a stir bar was charged with metallic lanthanum (0.93 g; 6.65 mmol), 2-methoxyethanol (10 mL), methanol (10 mL), and MnCl_2_ (0.42 g; 3.35 mmol). The mixture was vigorously stirred at room temperature for 68 h, and then the Schlenk tap was opened for 12 h to expose the reaction mixture for contact with air and moisture. The resulting dark brown solution was filtered off, concentrated under a vacuum, and left at room temperature for crystallization. Red crystals of **11** were grown at ambient temperature within 4 weeks. The crystals were filtered off, washed with hexane (3 × 5 mL), and dried under vacuum. Overall yield: 1.22 g (0.65 mmol, 39% calculated using MnCl_2_ as limiting reagent); Anal. Calcd for C_36_H_90_O_26_Cl_8_Mn_2_La_4_: C, 22.90; H, 4.80; Cl, 15.02; Mn, 5.82; La, 29.43. Found: C, 22.96; H, 4.82; Cl, 14.98; Mn, 5.87; La, 29.39. FTIR-ATR (cm^–1^): 3351 (m), 2933 (m), 1573 (vs), 1452 (m), 1359 (m), 1242 (w), 1197 (w), 1095 (m), 1046 (s), 1012 (m), 901 (w), 833 (w), 789 (vw), 494 (m).

### Synthesis of [Pr_4_Mn_2_(μ_3_-OH)_2_(μ_3_-OR)_4_(μ-OR)_4_(μ-Cl)_2_(HOR)_4_Cl_6_] (**12**)

A 150 mL Schlenk flask equipped with a stir bar was charged with metallic praseodymium (0.94 g; 6.65 mmol), 2-methoxyethanol (10 mL), methanol (10 mL), and MnCl_2_ (0.42 g; 3.35 mmol). The mixture was stirred at room temperature within 72 h, and then the Schlenk tap was opened for 8 h to expose the reaction to contact with air and moisture. The resulting dark brown solution was filtered off, concentrated under a vacuum, and left at room temperature for crystallization. Red crystals of **12** were grown at ambient temperature within 4 weeks. The crystals were filtered off, washed with hexane (3 × 5 mL), and dried under vacuum. Overall yield: 1.12 g (0.59 mmol, 35% calculated using MnCl_2_ as limiting reagent); Anal. Calcd for C_36_H_90_O_26_Cl_8_Mn_2_Pr_4_: C, 22.80; H, 4.78; Cl, 14.96; Mn, 5.79; Pr, 29.72. Found: C, 22.87; H, 4.80; Cl, 14.88; Mn, 5.81; Pr, 29.76. FTIR-ATR (cm^–1^): 3380 (w), 2926 (m), 2857 (m), 1587 (w), 1453 (w), 1390 (w), 1360 (w), 1271 (vw), 1241 (w), 1195 (w), 1097 (m), 1045 (vs), 1009 (s), 907 (m), 828 (m), 569 (m), 494 (vw), 428 (w).

### Synthesis of [MnCl_2_(HOR)]*_n_* (**13**)

A 150 mL Schlenk flask with a stir bar was charged with 2-methoxyethanol (5 mL) and MnCl_2_ (0.42 g; 3.35 mmol). The mixture was stirred at room temperature within 4 h, then concentrated under vacuum and left at room temperature for crystallization. Colorless crystals of **13** were grown at ambient temperature within 1 week. The crystals were filtered off, washed with hexane (3 × 5 mL), and dried under vacuum. Overall yield: 0.51 g (2.51 mmol, 75%); Anal. Calcd for C_3_H_8_O_2_Cl_2_Mn: C, 17.84; H, 3.99; Cl, 35.11. Found: C, 17.88; H, 3.96; Cl, 35.02. FTIR-ATR (cm^–1^): 3313 (s), 3194 (s), 2945 (m), 2893 (w), 2839 (w), 2525 (vw), 2335 (vw), 2080 (vw), 1632 (m), 1605 (m), 1451 (m), 1407 (w), 1367 (w), 1301 (w), 1235 (w), 1195 (m), 1156 (vw), 1109 (m), 1090 (s), 1042 (vs), 1008 (m), 973 (w), 890 (m), 867 (w), 821 (m), 711 (vw), 642 (vw), 546 (w), 525 (w), 449 (vw).

### Synthesis of [NiCl_2_(HOR)_2_] (**14**)

A 150 mL Schlenk flask equipped with a stir bar was charged with 2-methoxyethanol (5 mL) and NiCl_2_ (0.52 g; 4.0 mmol). The mixture was stirred at room temperature within 3 h, then concentrated under vacuum and left at room temperature for crystallization. Light green crystals of **14** were grown at ambient temperature within 1 week. The crystals were filtered off, washed with hexane (3 × 5 mL), and dried under vacuum. Overall yield: 0.92 g (3.28 mmol, 82%); Anal. Calcd for C_6_H_16_O_4_Cl_2_Ni: C, 25.57; H, 5.72; Cl, 25.16. Found: C, 25.60; H, 5.73; Cl, 24.99. FTIR-ATR (cm^–1^): 3178 (w), 3025 (vw), 2983 (w), 2959 (w), 2933 (w), 2880 (w), 2831 (vw), 2752 (vw), 1648 (w), 1582 (w), 1451 (w), 1428 (w), 1381 (w), 1352 (w), 1266 (w), 1218 (w), 1184 (w), 1076 (vs), 1031 (vs), 1008 (vs), 898 (m), 829 (m), 708 (vs), 559 (w), 487 (vw), 410 (m).

## Supporting Information Available

IR spectroscopic and crystallographic data for **1**–**14**, and powder XRD and EDX data for synthesized oxide materials (PDF)
