# Supplementary material for: Heterometallic 3d–4f Alkoxide Precursors for the Synthesis of Binary Oxide Nanomaterials
Source: Inorg Chem. 2023 Jan 25;62(5):2197–212. doi: 10.1021/acs.inorgchem.2c03872 (PMC9906784; doi:10.1021/acs.inorgchem.2c03872)
Supplement: Supplementary file 1 — ic2c03872_si_001.pdf [file ic2c03872_si_001.pdf]

Supporting information for

Heterometallic 3d-4f alkoxide precursors for the synthesis of binary oxide nanomaterials

Rafał Petrus,*[a] Adrian Kowaliński,[a] Józef Utko,[a] Karolina Matuszak,[a] Tadeusz Lis,[b] and Piotr Sobota*[a]

[a]Faculty of Chemistry, Wrocław University of Science and Technology, 23 Smoluchowskiego, 50-370 Wrocław, Poland

[b]Faculty of Chemistry, University of Wrocław, 14 F. Joliot-Curie, 50-383 Wrocław, Poland

Corresponding author:

Dr. Rafał Petrus, rafal.petrus@pwr.edu.pl

Prof. Dr. Piotr Sobota, piotr.sobota@pwr.edu.pl

Contents

X-Ray Crystallography of **1-12**....................S2

X-Ray Crystallography of **13-14**....................S18, S19

IR spectra of **1-12**....................S10

IR spectra of **13-14**....................S18, S20

PXRD study of oxide materials....................S16, S19

TEM-EDS analysis of oxide materials....................S21

Crystallographic Data for Compounds 1-14.

Table S1. Crystal and data collection parameters for compounds **1-14**.

Crystal	**1**·2(ROH)	**2**·2(ROH)	**3**	**4**·ROH
Chemical formula	$C_{42}H_{104}Cl_6ILa_4Mn_2O_{29}$	$C_{90}H_{216}Cl_{17.48}I_{0.52}Mn_6Nd_{12}O_{63}$	$C_{24}H_{56}Cl_6Gd_4Mn_2O_{17}$	$C_{33}H_{80}Cl_6Co_2O_{23}Pr_4$
Formula Mass	2078.37	5052.79	1568.26	1739.17
Crystal system	Triclinic	Triclinic	Tetragonal	Triclinic
Space group	$P\bar{1}$	$P\bar{1}$	$I4_1/a$	$P\bar{1}$
a/Å	11.021 (2)	11.805 (3)	21.255 (5)	12.023 (4)
b/Å	12.542 (2)	12.197 (3)		12.449 (4)
c/Å	13.388 (3)	28.713 (6)	20.616 (6)	20.674 (7)
α/°	99.65 (2)	85.83 (3)		102.11 (3)
β/°	90.05 (2)	86.40 (3)		103.33 (3)
γ/°	95.79 (3)	81.79 (3)		95.25 (3)
Unit cell volume/Å^3	1814.8 (6)	4075.3 (17)	9314 (5)	2912.0 (17)
Temperature/K	100(2)	80(2)	90(2)	100(2)
Z	1	1	8	2
Radiation type	MoKα	MoKα	MoKα	MoKα
Absorption coefficient, μ/mm^{-1}	3.357	4.653	6.543	4.180
No. of reflections measured	21989	27727	24181	31121
No. of independent reflections	7498	15617	6662	10798
No. of observed reflections ($I > 2\sigma(I)$)	6642	12686	5659	9143
R_{int}	0.0330	0.0302	0.0347	0.0362
Final R_1 values ($I > 2\sigma(I)$)	0.0382	0.0353	0.0234	0.0715
Final $wR(F^2)$ values ($I > 2\sigma(I)$)	0.1064	0.0670	0.0401	0.1495
Final R_1 values (all data)	0.0427	0.0506	0.0335	0.0860
Final $wR(F^2)$ values (all data)	0.1101	0.0725	0.0424	0.1558
Goodness of fit on F^2	1.05	1.03	1.03	1.20
Δρmax/eÅ^{-3}	1.75	1.83	0.69	2.70
Δρmin/eÅ^{-3}	-1.74	-1.20	-0.51	-2.07

Crystal	**4**·0.5(ROH) 0.25(EtOH)	**5**	**6**	**7**
Chemical formula	$C_{34}H_{85.5}Cl_6Co_2O_{22.25}Pr_4$	$C_{36}H_{88}Cl_6Ni_2O_{25}Pr_4$	$C_6H_{15}Cl_2O_4Pr$	$C_{24}H_{60}Cl_4Co_4O_{16}$
Formula Mass	1744.72	1814.82	362.99	982.24
Crystal system	Monoclinic	Triclinic	Triclinic	Monoclinic
Space group	$P2_1/n$	$P\bar{1}$	$P\bar{1}$	$P2_1/c$
a/Å	13.407 (3)	12.253 (4)	6.694 (2)	14.555 (3)
b/Å	23.014 (4)	12.424 (4)	9.337 (2)	12.428 (2)
c/Å	19.349 (4)	12.533 (4)	10.331 (2)	23.581 (5)
α/°		112.64 (3)	108.15 (3)	
β/°	90.94 (2)	99.00 (3)	107.26 (3)	103.89 (3)
γ/°		113.46 (3)	99.34 (3)	
Unit cell volume/Å^3	5969 (2)	1503.4 (10)	562.4 (3)	4140.8 (15)
Temperature/K	100(2)	100(2)	100(2)	240(2)
Z	4	1	2	4
Radiation type	MoKα	MoKα	MoKα	MoKα
Absorption coefficient, μ/mm^{-1}	4.078	4.130	4.788	1.892
No. of reflections measured	63505	12048	7606	21744
No. of independent reflections	15092	6503	2457	10732
No. of observed reflections ($I > 2\sigma(I)$)	12511	6238	2250	8746
R_{int}	0.0331	0.0131	0.0333	0.0262
Final R_1 values ($I > 2\sigma(I)$)	0.0267	0.0163	0.0210	0.0307
Final $wR(F^2)$ values ($I > 2\sigma(I)$)	0.0506	0.0391	0.0436	0.0616
Final R_1 values (all data)	0.0385	0.0176	0.0246	0.0450
Final $wR(F^2)$ values (all data)	0.0546	0.0395	0.0447	0.0675
Goodness of fit on F^2	1.07	1.16	1.04	1.02
Δρmax/eÅ^{-3}	1.52	0.47	0.69	0.42
Δρmin/eÅ^{-3}	-0.93	-0.67	-0.48	-0.51

Crystal	**8**	**9**	**10**	**11**·2(ROH)
Chemical formula	$C_{20}H_{52}Cl_4Ni_4O_{12}$	$C_{22}H_{56}Cl_4Mn_4O_{14}$	$C_{12}H_{32}ClNdO_8Cl_4Co$	$C_{42}H_{106}Cl_8La_4Mn_2O_{30}$
Formula Mass	861.25	906.22	684.79	2040.38
Crystal system	Monoclinic	Monoclinic	Monoclinic	Triclinic
Space group	$P2_1/c$	$P2_1/c$	$P2_1/c$	$P\bar{1}$
a/Å	12.701 (2)	13.062 (2)	14.1794 (11)	10.7955 (18)
b/Å	12.165 (2)	12.404 (2)	10.3133 (8)	13.221 (2)
c/Å	23.118 (3)	23.631 (3)	16.760 (2)	13.327 (2)
α/°				95.45 (3)
β/°	91.07 (2)	91.35 (2)	91.78 (2)	97.89 (3)
γ/°				90.91 (3)
Unit cell volume/Å^3	3571.3 (9)	3827.7 (10)	2449.7 (4)	1874.8 (5)
Temperature/K	100(2)	100(2)	100(2)	100(2)
Z	4	4	4	1
Radiation type	MoKα	MoKα	MoKα	MoKα
Absorption coefficient, μ/mm^{-1}	2.42	1.623	3.350	2.912
No. of reflections measured	84220	89302	39665	18953
No. of independent reflections	7799	8344	7225	7365
No. of observed reflections ($I > 2\sigma(I)$)	7360	7458	6994	6867
R_{int}	0.0421	0.036	0.019	0.040
Final R_1 values ($I > 2\sigma(I)$)	0.0201	0.0308	0.0145	0.0363
Final $wR(F^2)$ values ($I > 2\sigma(I)$)	0.0521	0.0853	0.0332	0.1015
Final R_1 values (all data)	0.0219	0.0358	0.0155	0.0382
Final $wR(F^2)$ values (all data)	0.0539	0.0897	0.0335	0.1032
Goodness of fit on F^2	1.05	1.08	1.04	1.05
Δρmax/eÅ^{-3}	1.31	0.56	0.85	1.44
Δρmin/eÅ^{-3}	-0.40	-0.82	0.44	-1.40

Crystal	**12**	**13**	**14**
Chemical formula	$C_{42}H_{106}Cl_8Mn_2O_{30}Pr_4$	$C_{27}H_{72}Cl_{18}Mn_9O_{18}$	$C_6H_{16}Cl_2NiO_4$
Formula Mass	2048.38	1817.40	281.80
Crystal system	Triclinic	Monoclinic	Monoclinic
Space group	$P\bar{1}$	$P2_1/c$	$C2/c$
a/Å	10.760 (2)	10.9932 (18)	13.275 (3)
b/Å	13.249 (3)	45.707 (9)	10.086 (2)
c/Å	13.274 (3)	13.360 (3)	9.915 (2)
α/°	86.21 (2)		
β/°	81.86 (2)	106.96 (3)	119.01 (5)
γ/°	88.30 (2)		
Unit cell volume/Å^3	1868.8 (7)	6421 (2)	1161.0 (6)
Temperature/K	100(2)	100(2)	100(2)
Z	1	4	4
Radiation type	MoKα	MoKα	MoKα
Absorption coefficient, μ/mm^{-1}	3.242	2.512	2.114
No. of reflections measured	25525	56386	2708
No. of independent reflections	8136	12601	1234
No. of observed reflections ($I > 2\sigma(I)$)	7674	7939	1082
R_{int}	0.0162	0.0568	0.0383
Final R_1 values ($I > 2\sigma(I)$)	0.0275	0.0608	0.0429
Final $wR(F^2)$ values ($I > 2\sigma(I)$)	0.0714	0.1128	0.1103
Final R_1 values (all data)	0.0293	0.1025	0.0492
Final $wR(F^2)$ values (all data)	0.0726	0.1288	0.1169
Goodness of fit on F^2	1.08	1.12	1.10
Δρmax/eÅ^{-3}	0.85	1.13	0.64
Δρmin/eÅ^{-3}	-0.82	-0.58	-0.76

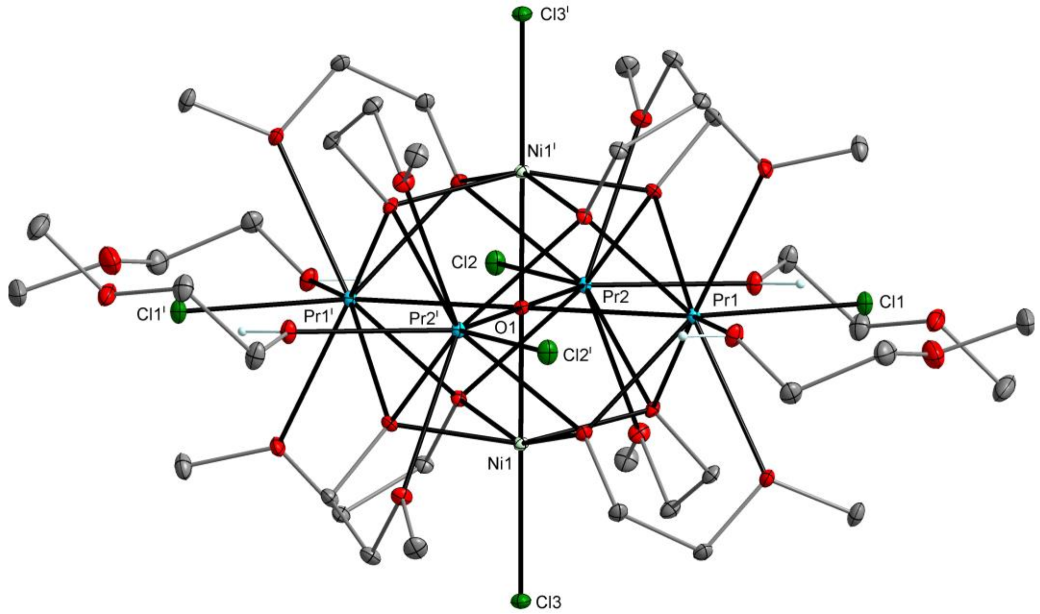

Figure S1. Molecular structure of $[Pr_4Ni_2(\mu_6\text{-}O)(\mu_3\text{-}OR)_8(HOR)_4Cl_6]$ (**5**). Displacement ellipsoids are drawn at the 30% probability level. The hydrogen atoms of the alkyl groups are omitted for clarity [symmetry code: (i) −*x*+1, −*y*+1, −*z*+1].

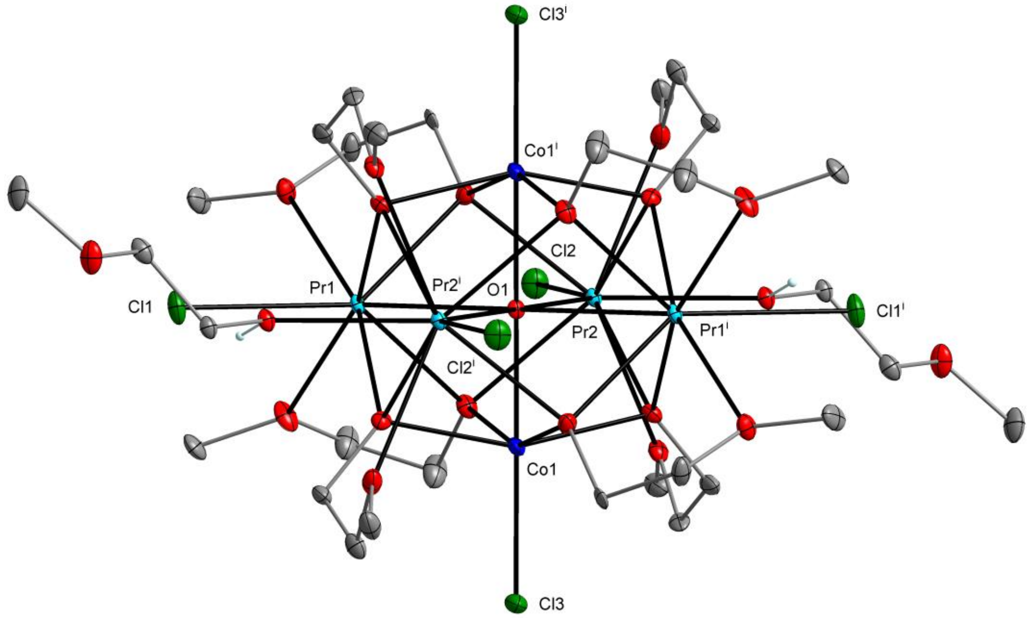

Figure S2. Molecular structure of $[Pr_4Co_2(\mu_6\text{-}O)(\mu_3\text{-}OR)_8(HOR)_2Cl_6]\cdot ROH$ (**4**). Displacement ellipsoids are drawn at the 20% probability level. The hydrogen atoms of the alkyl groups and the second part of the disordered praseodymium atoms are omitted for clarity [symmetry code: (i) −*x*+1, −*y*, −*z*].

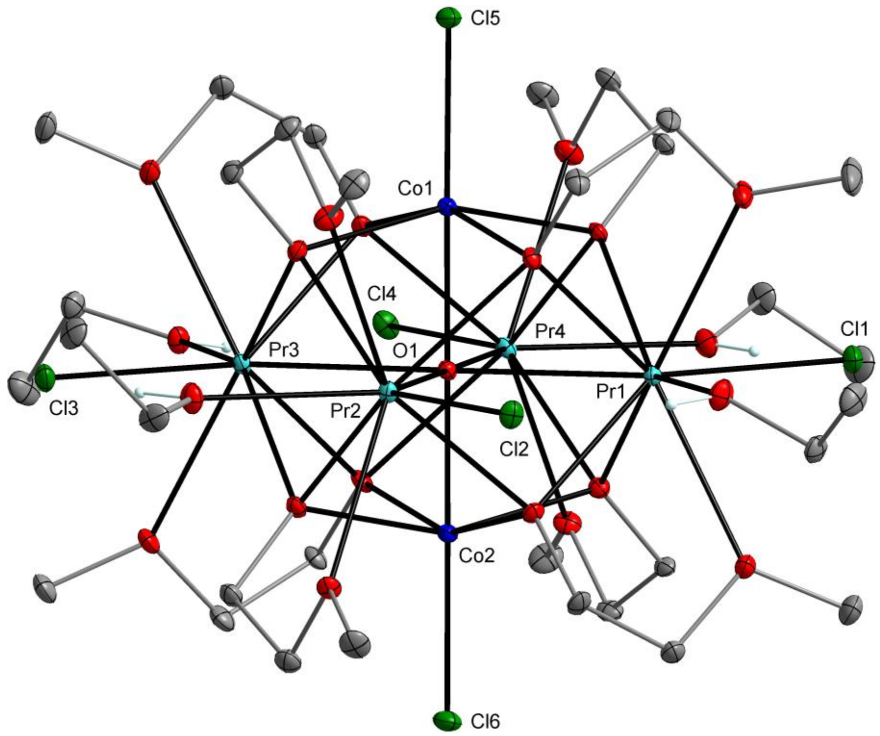

Figure S3. Molecular structure of $[Pr_4Co_2(\mu_6\text{-}O)(\mu_3\text{-}OR)_8(EtOH)_4Cl_6]\cdot EtOH$ (**4a**). Displacement ellipsoids are drawn at the 30% probability level. The hydrogen atoms of the alkyl groups are omitted for clarity.

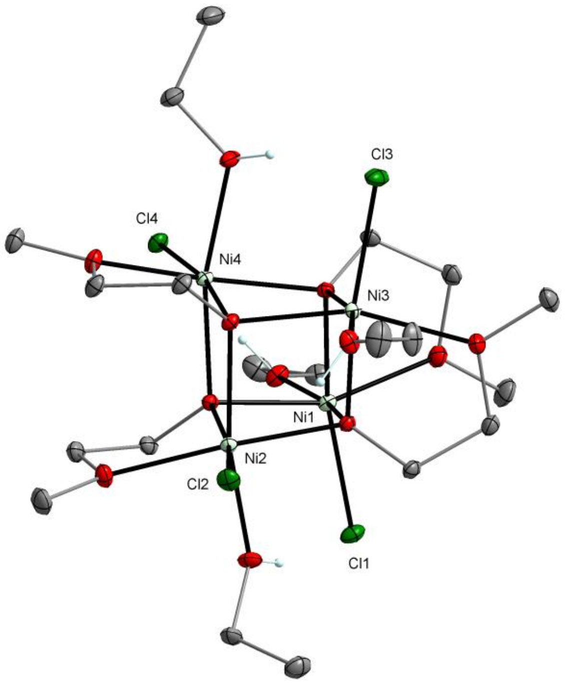

Figure S4. Molecular structure of $[Ni_4(\mu_3\text{-}OR)_4(HOEt)_4Cl_4]$ (**8**). Displacement ellipsoids are drawn at the 30% probability level. The hydrogen atoms of the alkyl groups are omitted for clarity.

Table S2. Continuous-shape measurements (CShM) of the coordination environment around metal ions in **1-5** and **11-12**.

compound	atom	donor atoms	polyhedron	*S* parameter
**1**	La1	O_8Cl	gyroelongated square pyramid J10	1.633
	La2	O_8Cl	gyroelongated square pyramid J10	1.437
	Mn1	O_5Cl	octahedron	1.802
	Mn2	O_5Cl	octahedron	2.621
**2**	Nd1	O_8Cl	capped square antiprism	1.287
	Nd2	O_7Cl	Johnson - biaugmented trigonal prism (J50)	2.207
	Nd3	O_7Cl	biaugmented trigonal prism	2.397
	Nd4	O_7Cl	biaugmented trigonal prism	2.009
	Nd5	O_7Cl	Johnson - biaugmented trigonal prism (J50)	2.522
	Nd6	O_8Cl	capped square antiprism	1.714
	Mn1	O_5Cl	octahedron	2.621
	Mn2	O_5Cl	octahedron	2.749
	Mn3	O_5Cl	octahedron	2.721
**3**	Gd1	O_7Cl	biaugmented trigonal prism	2.047
	Gd2	O_7Cl	biaugmented trigonal prism	1.590
	Mn1	O_5Cl	octahedron	2.989
**4**	Pr1	O_7Cl	Johnson - biaugmented trigonal prism (J50)	2.461
	Pr2	O_7Cl_2	capped square antiprism	1.384
	Pr3	O_7Cl_2	capped square antiprism	1.283
	Pr4	O_7Cl	Johnson - biaugmented trigonal prism (J50)	2.490
	Co1	O_5Cl	octahedron	1.866
	Co2	O_5Cl	octahedron	1.903
**4a**	Pr1	O_8Cl	capped square antiprism	1.312
	Pr2	O_8Cl	capped square antiprism	1.266
	Pr3	O_8Cl	capped square antiprism	1.225
	Pr4	O_8Cl	capped square antiprism	1.237
	Co1	O_5Cl	octahedron	1.882
	Co2	O_5Cl	octahedron	1.854
**5**	Pr1	O_8Cl	capped square antiprism	1.273
	Pr2	O_8Cl	capped square antiprism	1.286
	Ni1	O_5Cl	octahedron	1.655
**11**	La1	O_7Cl_2	capped square antiprism	1.720
	La2	O_7Cl_2	muffin	1.123
	Mn1	O_5Cl	octahedron	2.189
**12**	Pr1	O_7Cl_2	capped square antiprism	1.490
	Pr2	O_7Cl_2	muffin	1.031
	Mn1	O_5Cl	octahedron	2.224

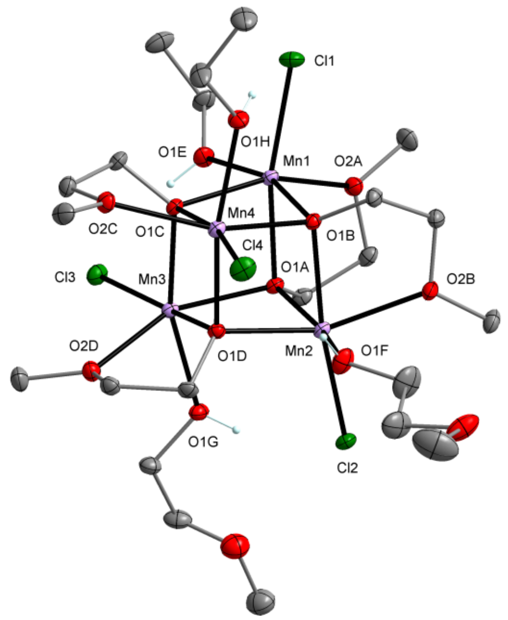

Figure S5. Molecular structure of $[Mn_4(\mu_3\text{-}OR)_4(HOR)_2(HOEt)_2Cl_4]$ (**9**). Displacement ellipsoids are drawn at the 30% probability level. The hydrogen atoms of the alkyl groups and the second and third parts of the disordered alkoxy ligands are omitted for clarity.

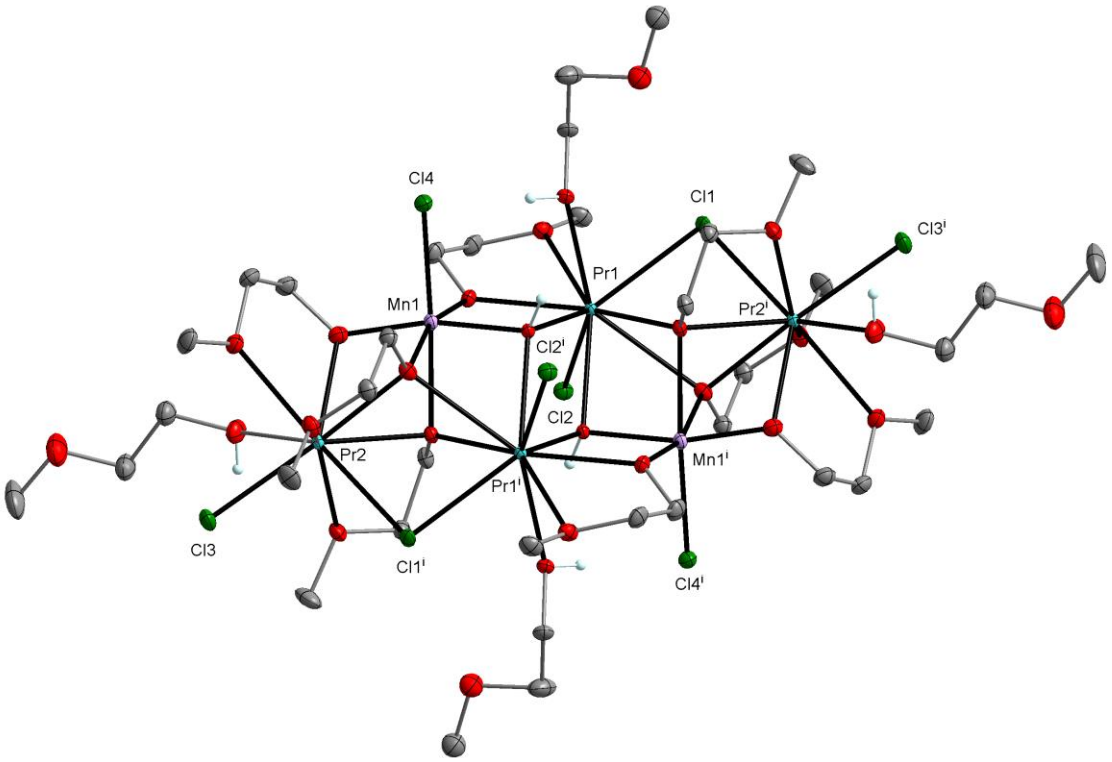

Figure S6. Molecular structure of $[Pr_4Mn_2(\mu_3\text{-}OH)_2(\mu_3\text{-}OR)_4(\mu\text{-}OR)_4(\mu\text{-}Cl)_2(HOR)_4Cl_6]$ (**12**). Displacement ellipsoids are drawn at the 20% probability level. The hydrogen atoms of the alkyl groups and the second part of the disordered praseodymium atoms and alkoxy ligands are omitted for clarity [symmetry code: (i) $-x+1$, $-y+1$, $-z+1$].

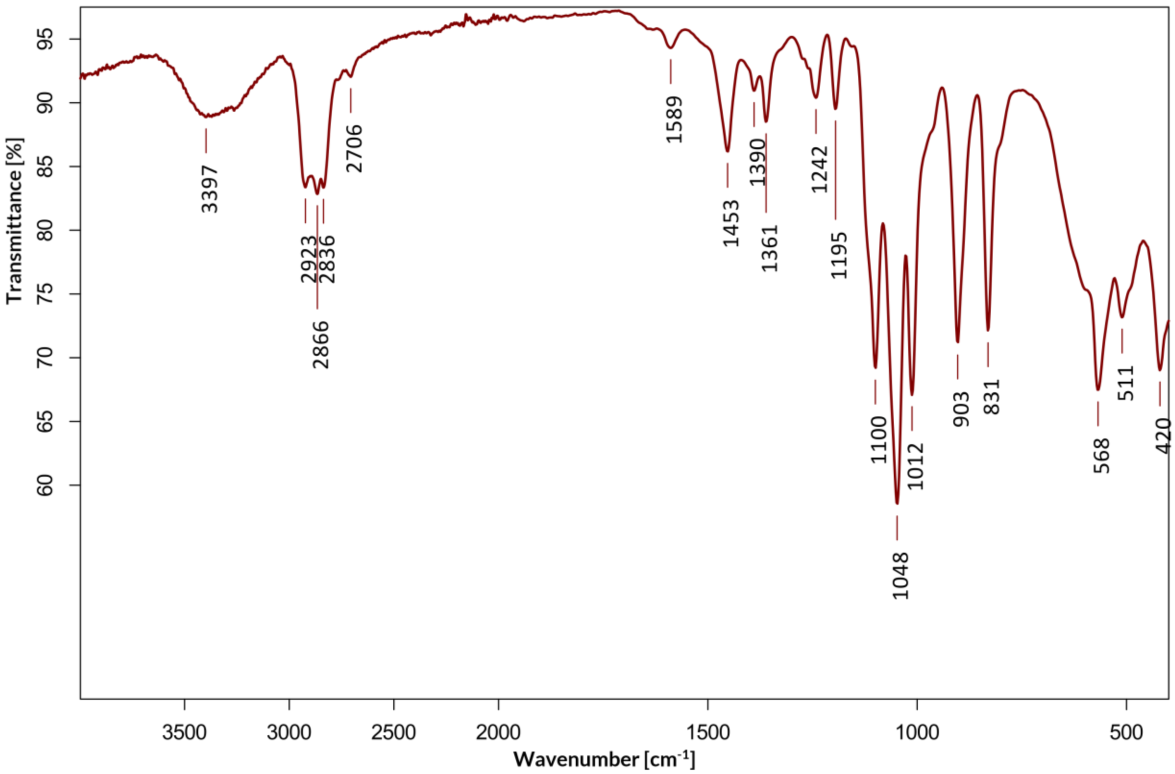

Figure S7. FTIR-ATR spectrum of **1**.

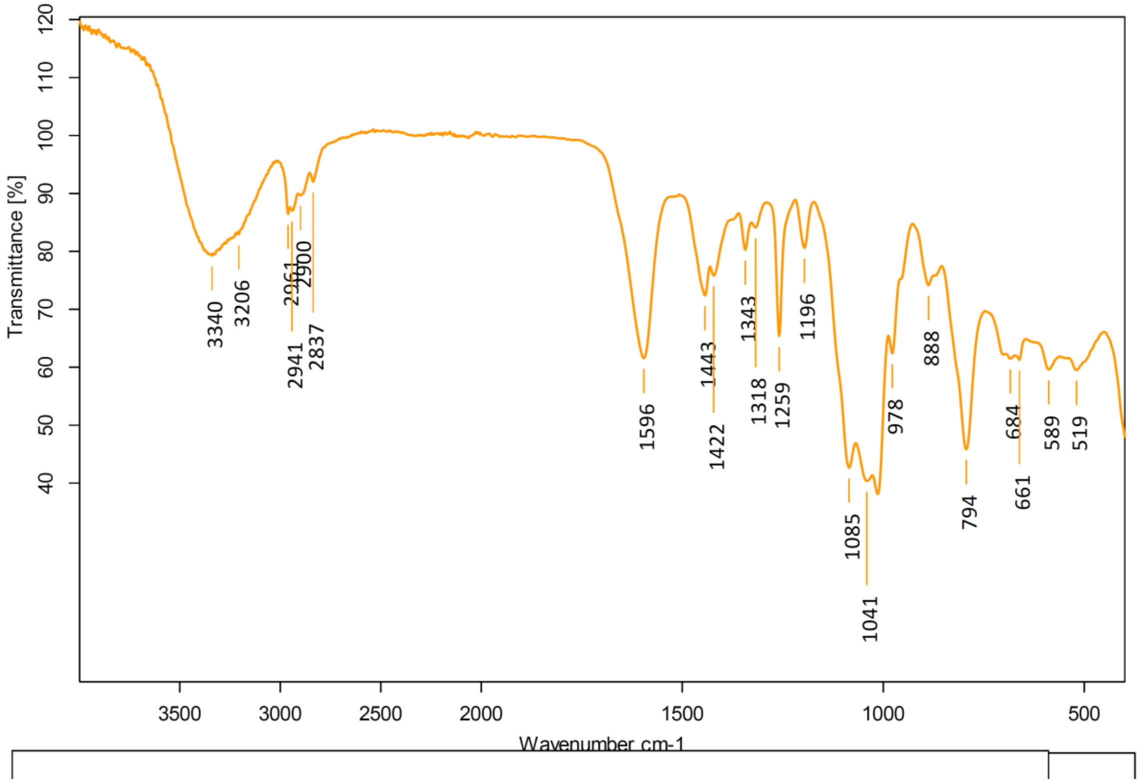

Figure S8. FTIR-ATR spectrum of **2**.

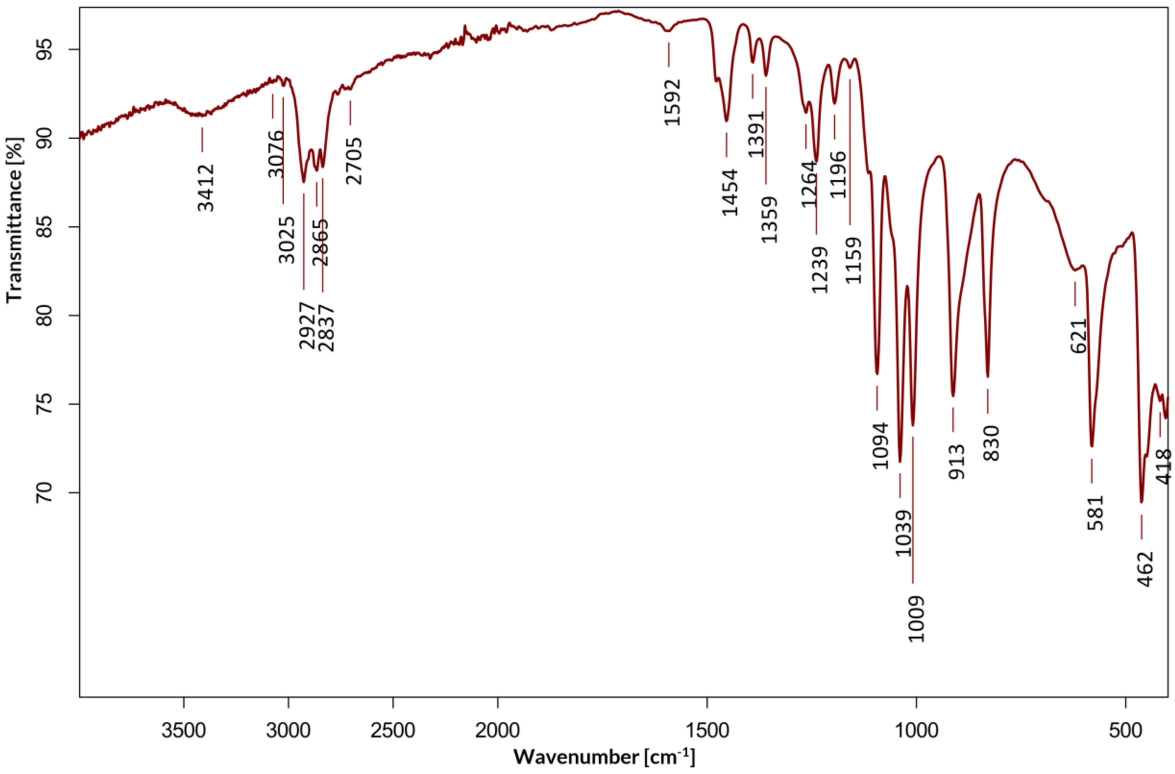

Figure S9. FTIR-ATR spectrum of **3**.

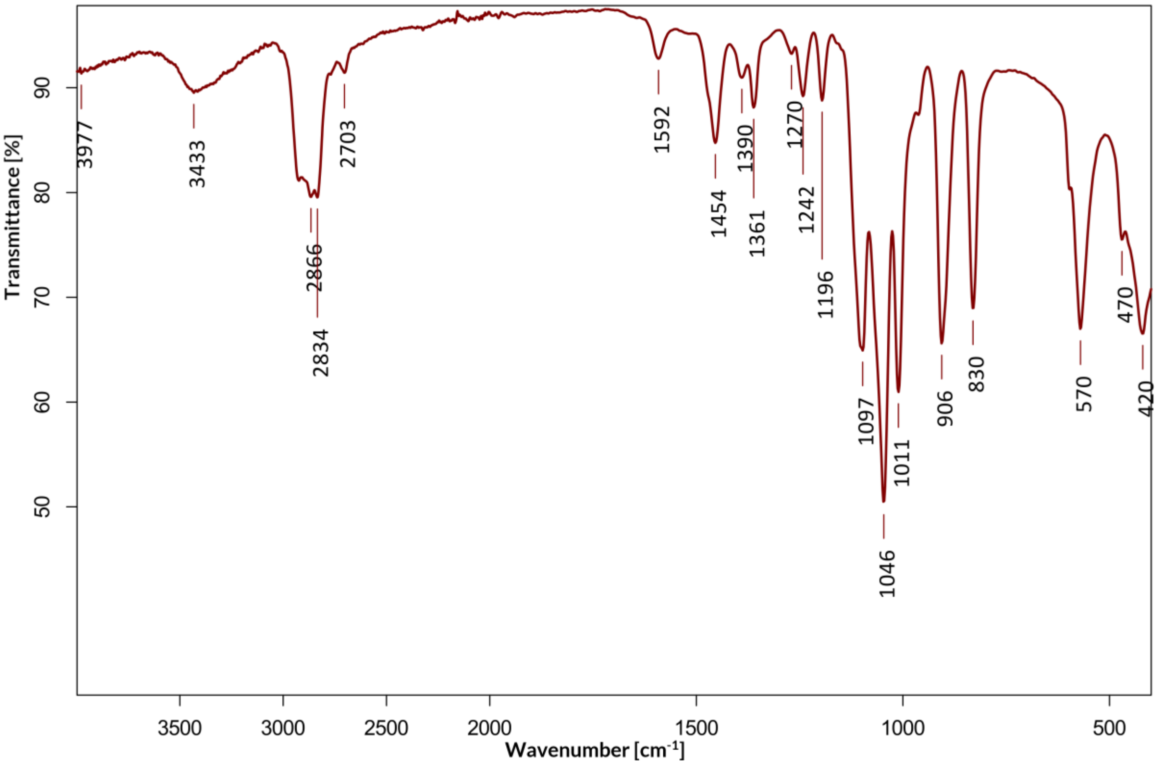

Figure S10. FTIR-ATR spectrum of **4**.

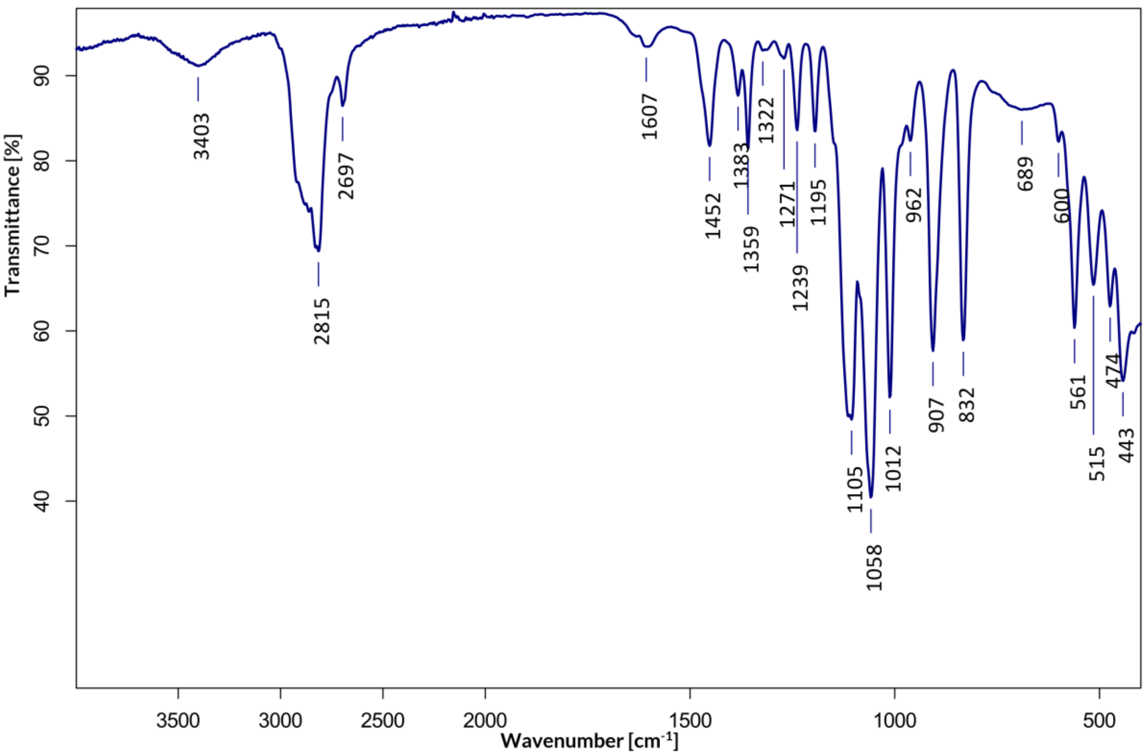

Figure S11. FTIR-ATR spectrum of **5**.

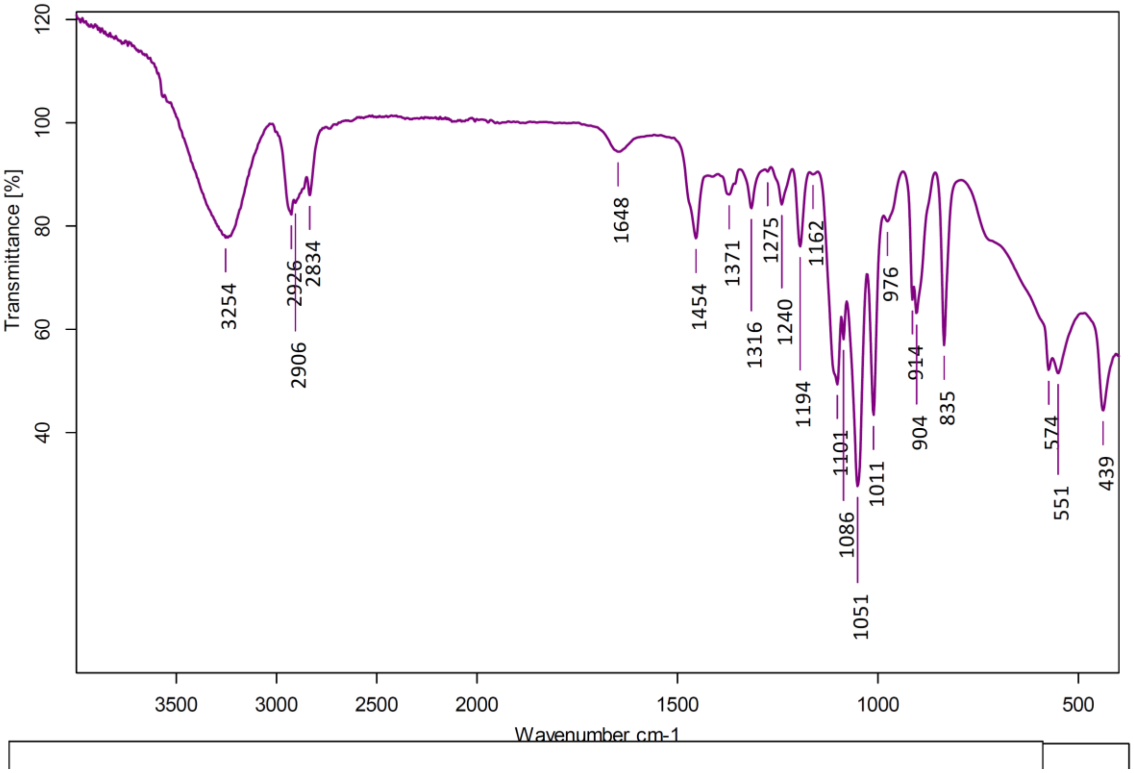

Figure S12. FTIR-ATR spectrum of **6**.

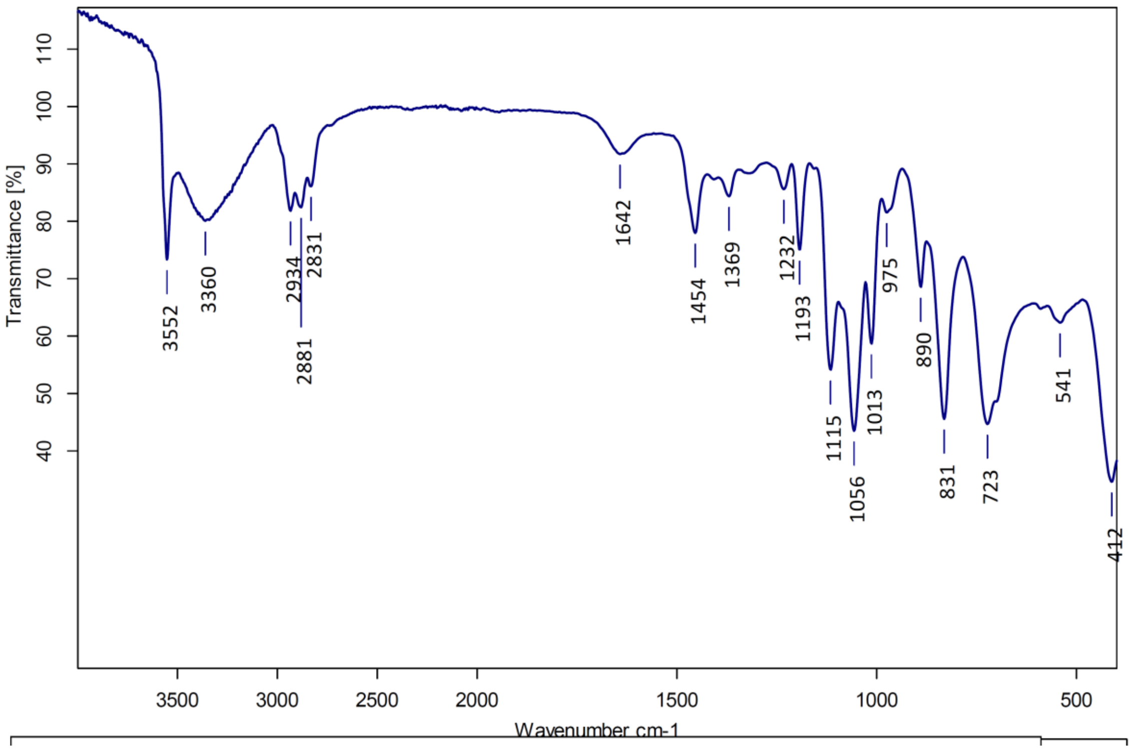

Figure S13. FTIR-ATR spectrum of **7**.

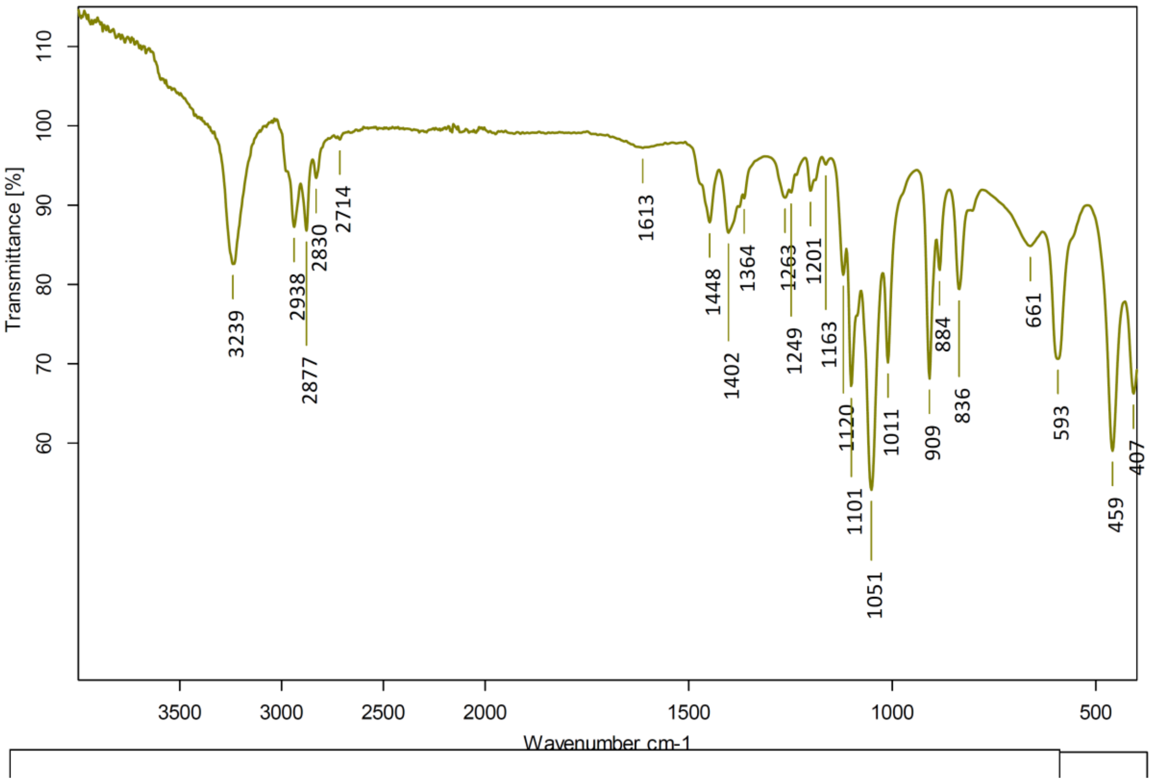

Figure S14. FTIR-ATR spectrum of **8**.

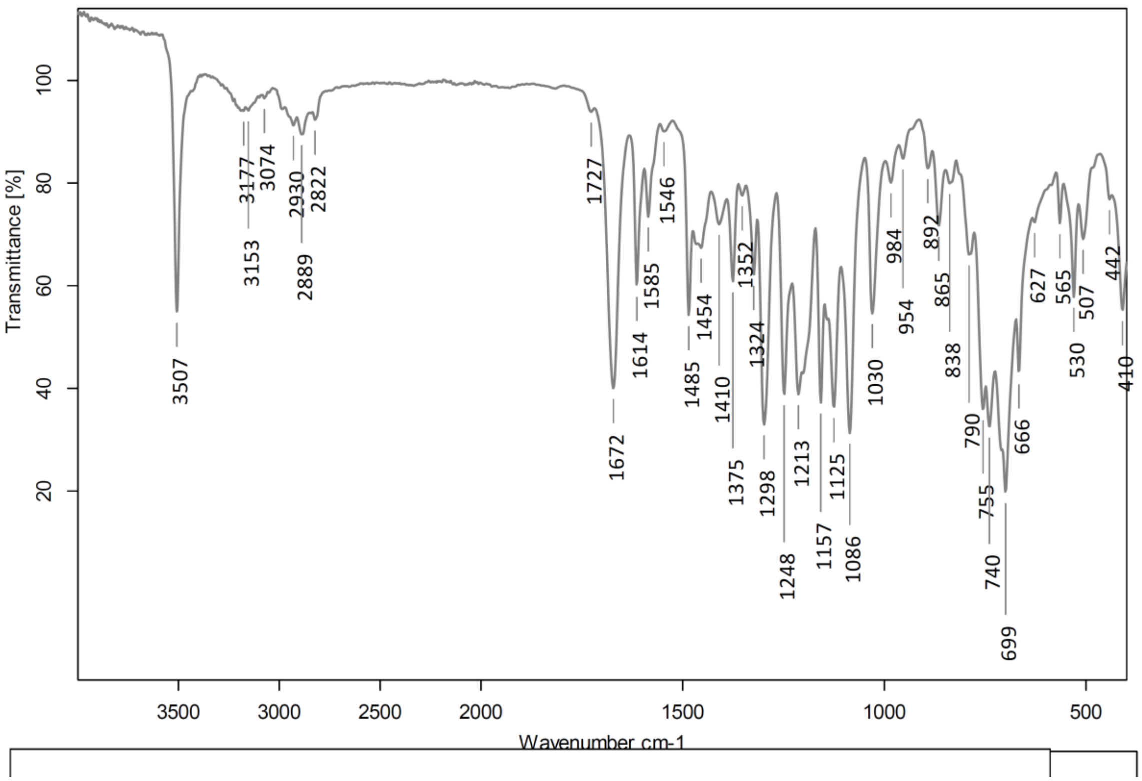

Figure S15. FTIR-ATR spectrum of **9**.

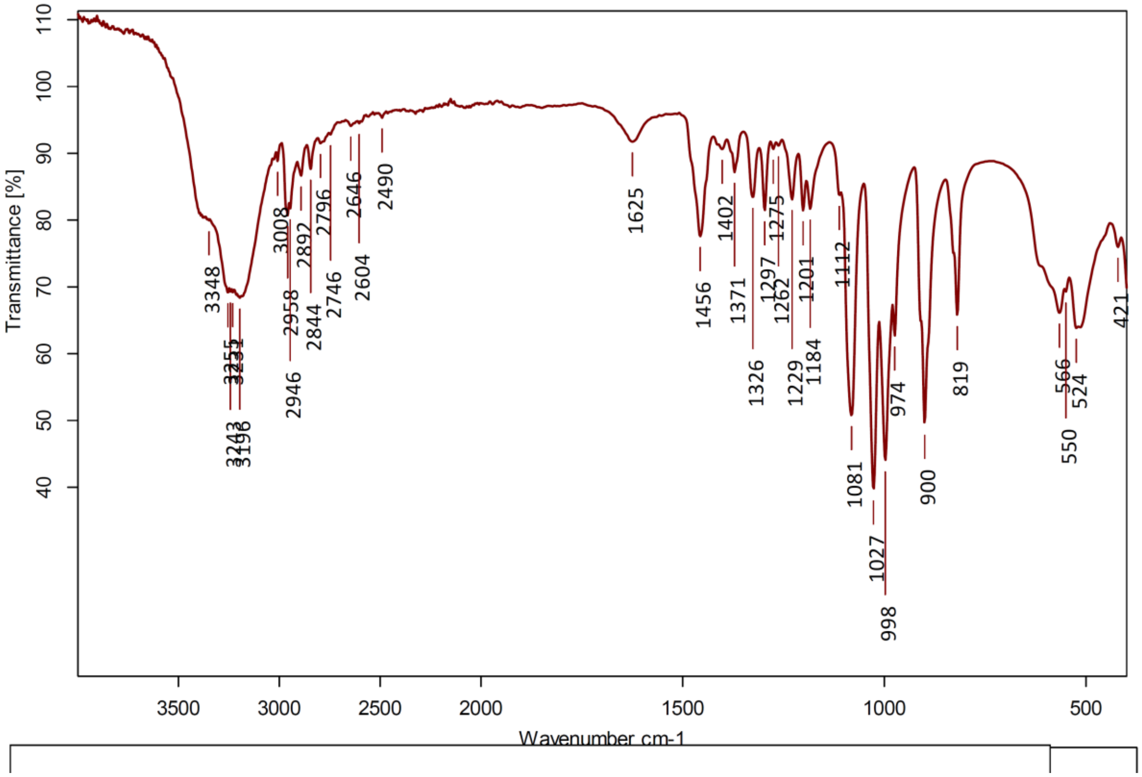

Figure S16. FTIR-ATR spectrum of **10**.

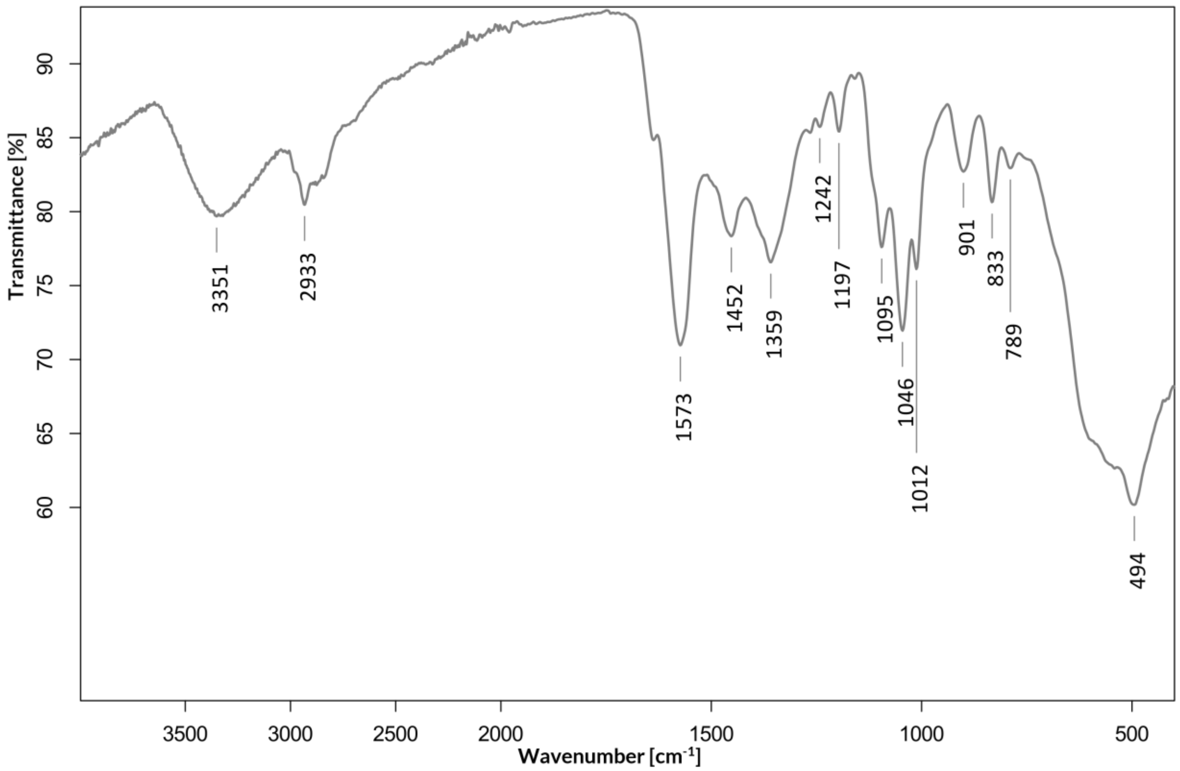

Figure S17. FTIR-ATR spectrum of **11**.

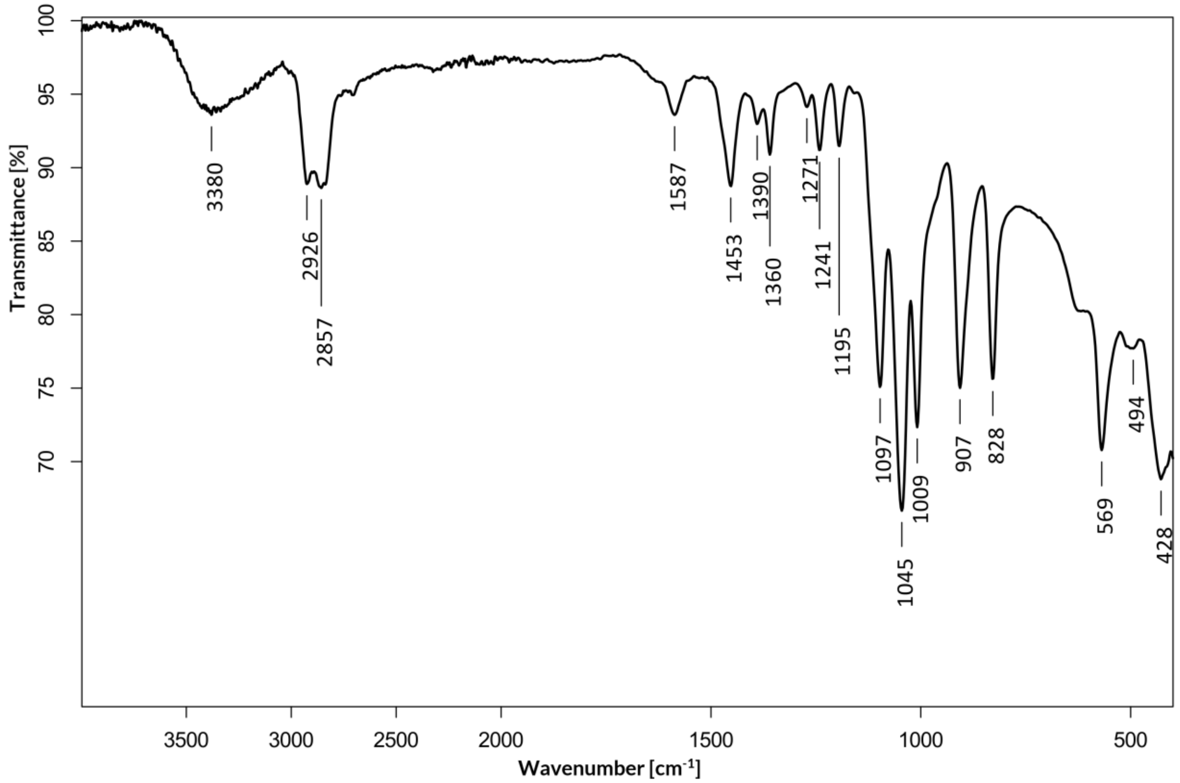

Figure S18. FTIR-ATR spectrum of **12**.

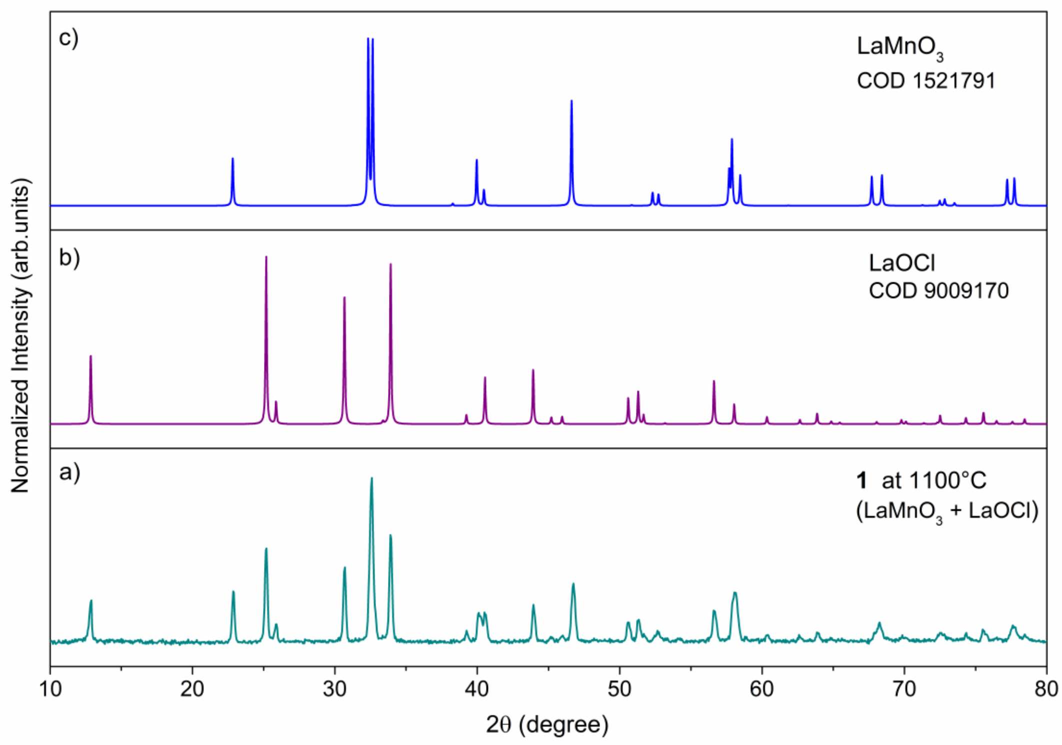

Figure S19. PXRD patterns of oxide materials prepared by calcination of **1** at 1100 °C (a), LaOCl [COD 2021; 9009170] (b), $LaMnO_3$ [1521791] (c).

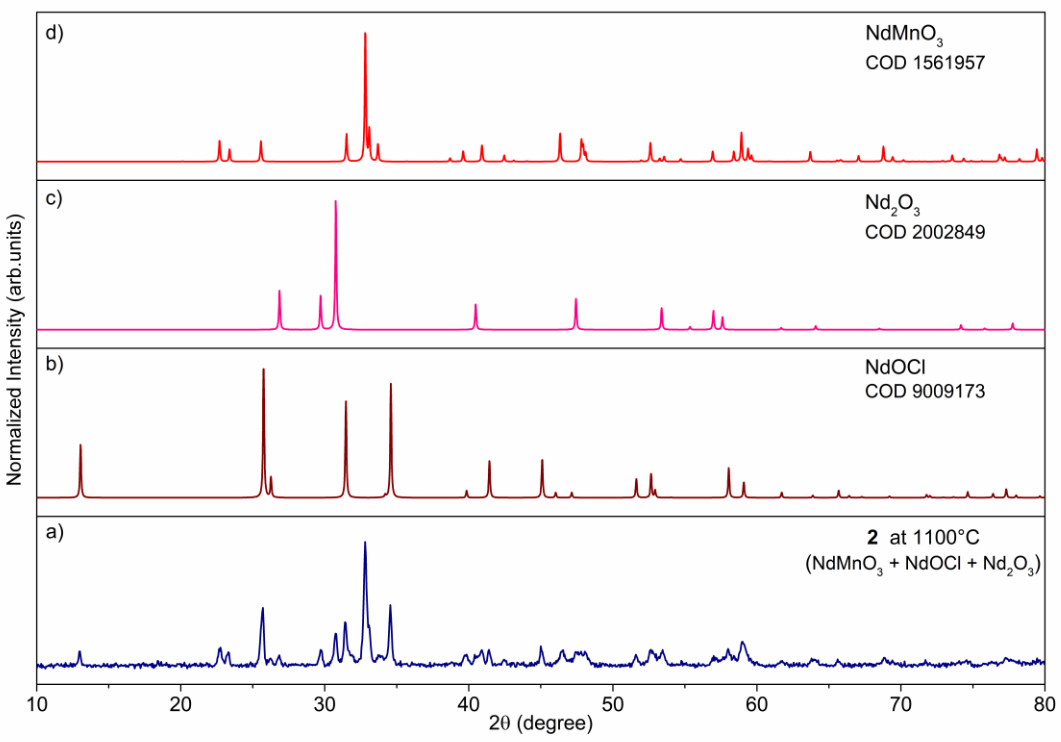

Figure S20. PXRD patterns of oxide materials prepared by calcination of **2** at 1100 °C (a), NdOCl [COD 2021; 9009173] (b), Nd_2O_3 [2002849] (c), $NdMnO_3$ [1561957] (d).

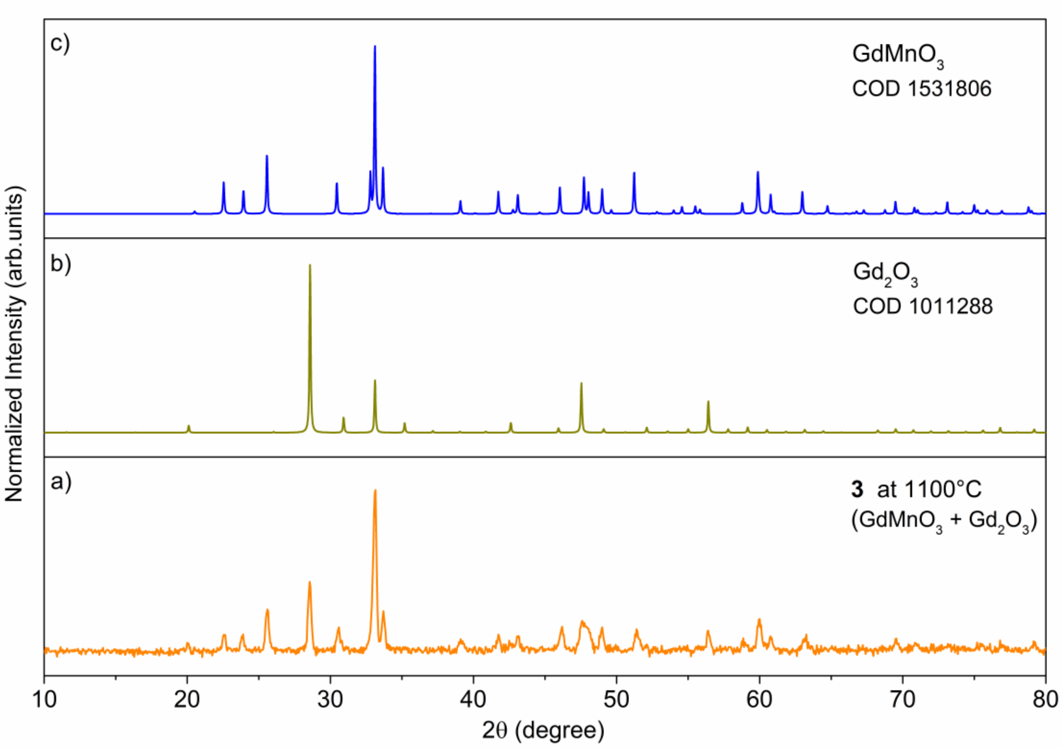

Figure S21. PXRD patterns of oxide materials prepared by calcination of **3** at 1100 °C (a), Gd_2O_3 [COD 2021; 1011288] (b), $GdMnO_3$ [1531806] (c).

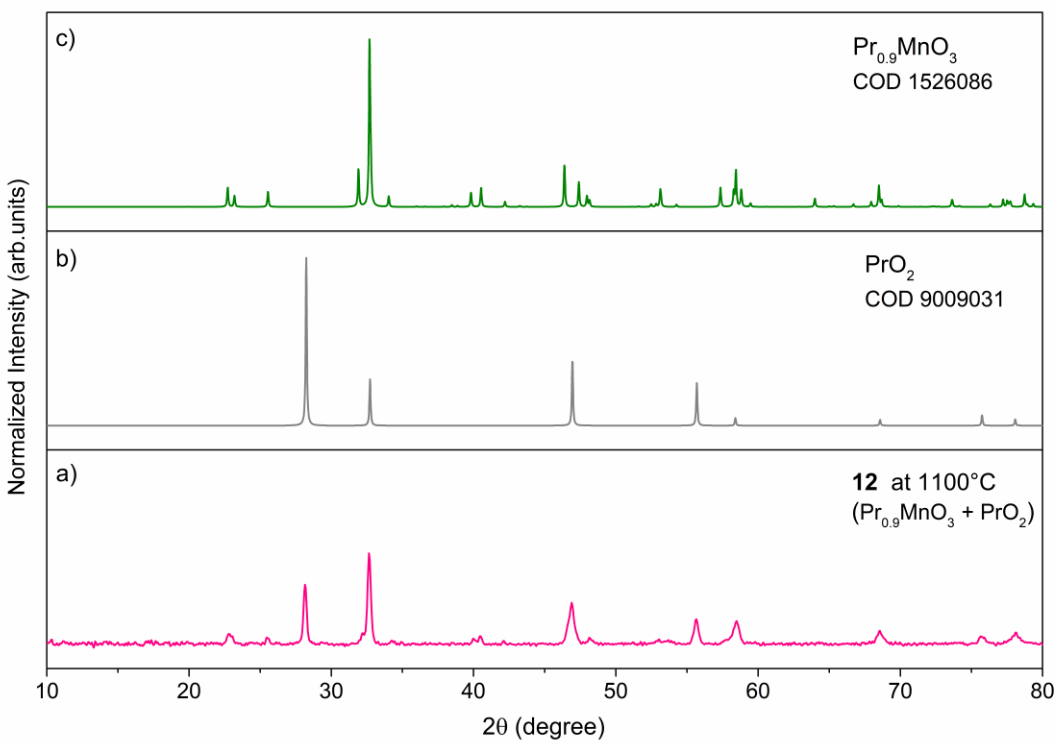

Figure S22. PXRD patterns of oxide materials prepared by calcination of **12** at 1100 °C (a), PrO_2 [COD 2021; 9009031] (b), $Pr_{0.9}MnO_3$ [1526086] (c).

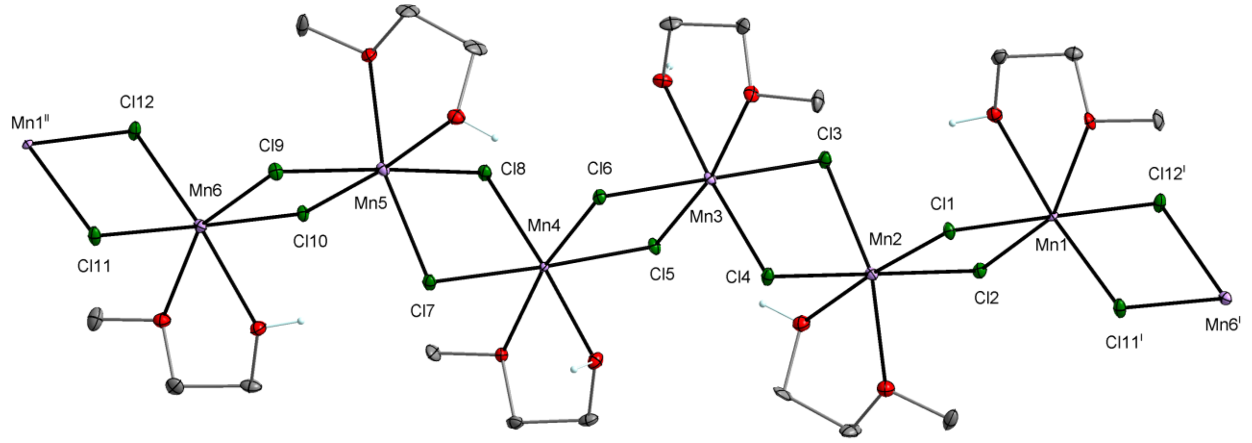

Figure S23. Molecular structure of [$MnCl_2(HOR)]_n$ (**13**). Displacement ellipsoids are drawn at the 30% probability level. The hydrogen atoms of the alkyl groups and the second part of the disordered manganese atoms are omitted for clarity [symmetry code: (i) $x-1$, y, $z+1$; (ii) $x+1$, y, $z-1$].

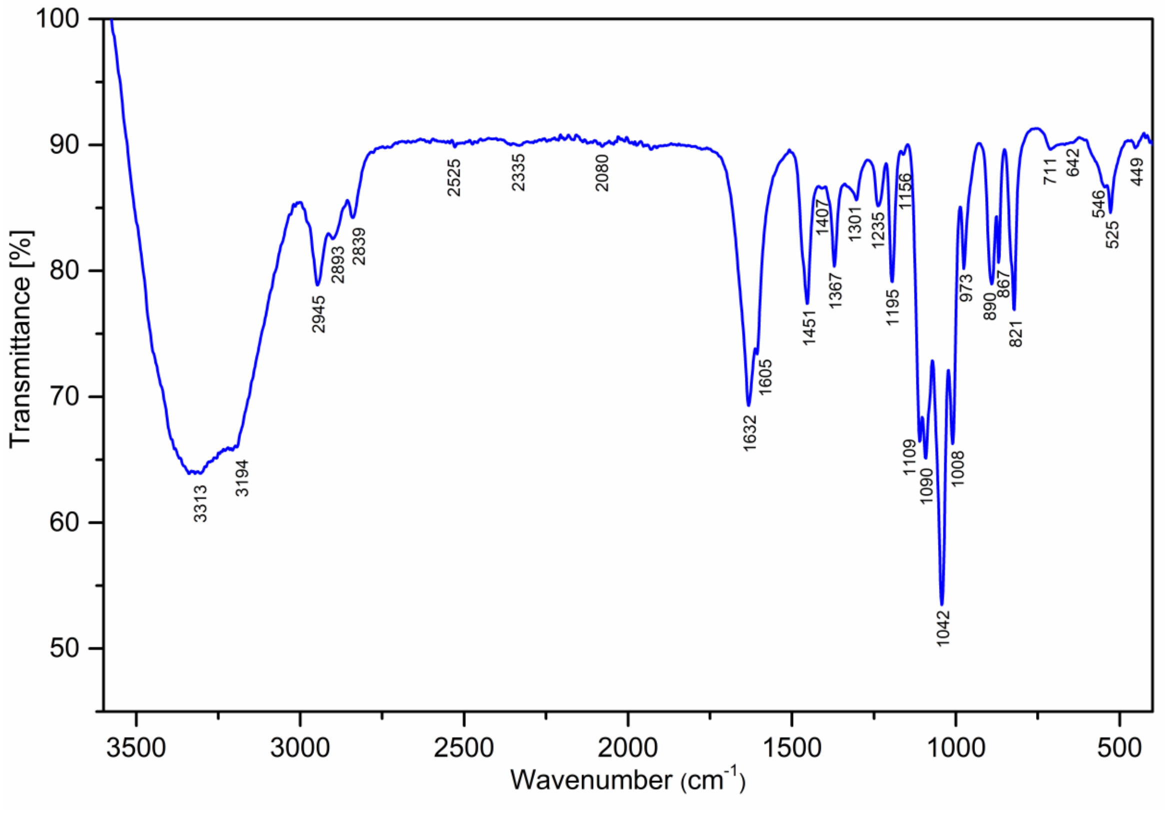

Figure S24. FTIR-ATR spectrum of **13**.

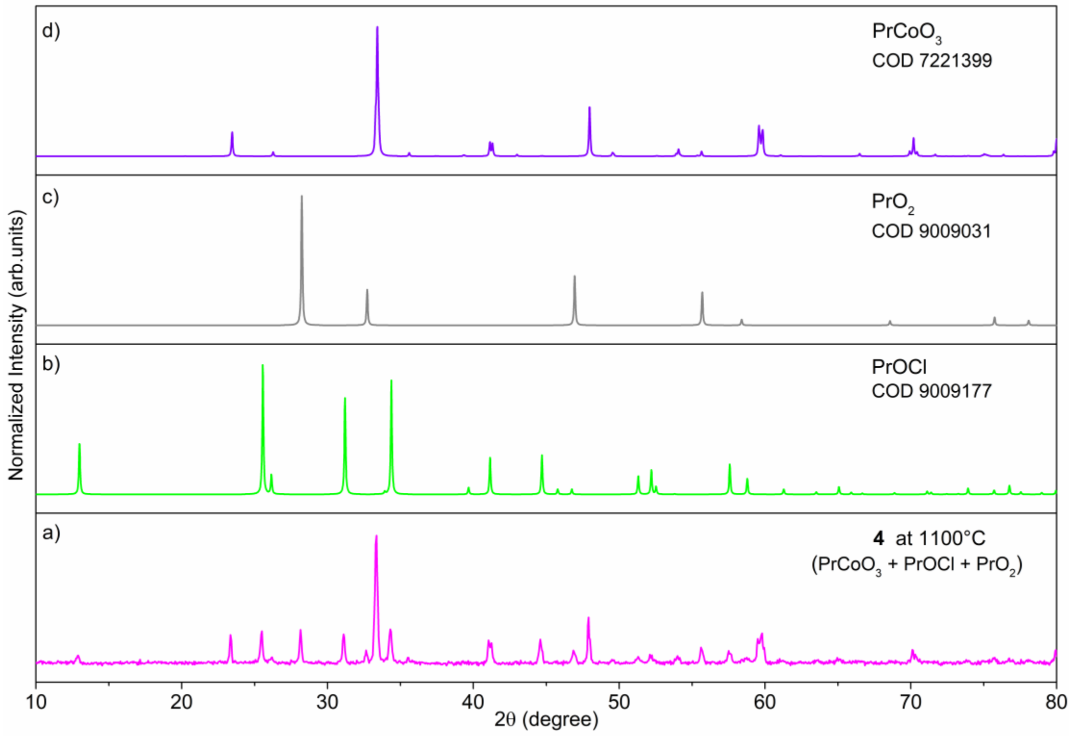

Figure S25. PXRD patterns of oxide materials prepared by calcination of **4** at 1100 °C (a), PrOCl [COD 2021; 9009177] (b), PrO_2 [9009031] (c), $PrCoO_3$ [7221399] (d).

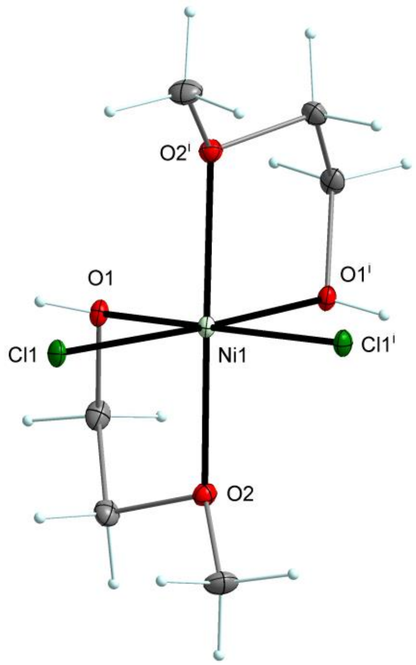

Figure S26. Molecular structure of [$NiCl_2$(HOR)] (**14**). Displacement ellipsoids are drawn at the 30% probability level [symmetry code: (i) −*x*+1, *y*, −*z*+1/2].

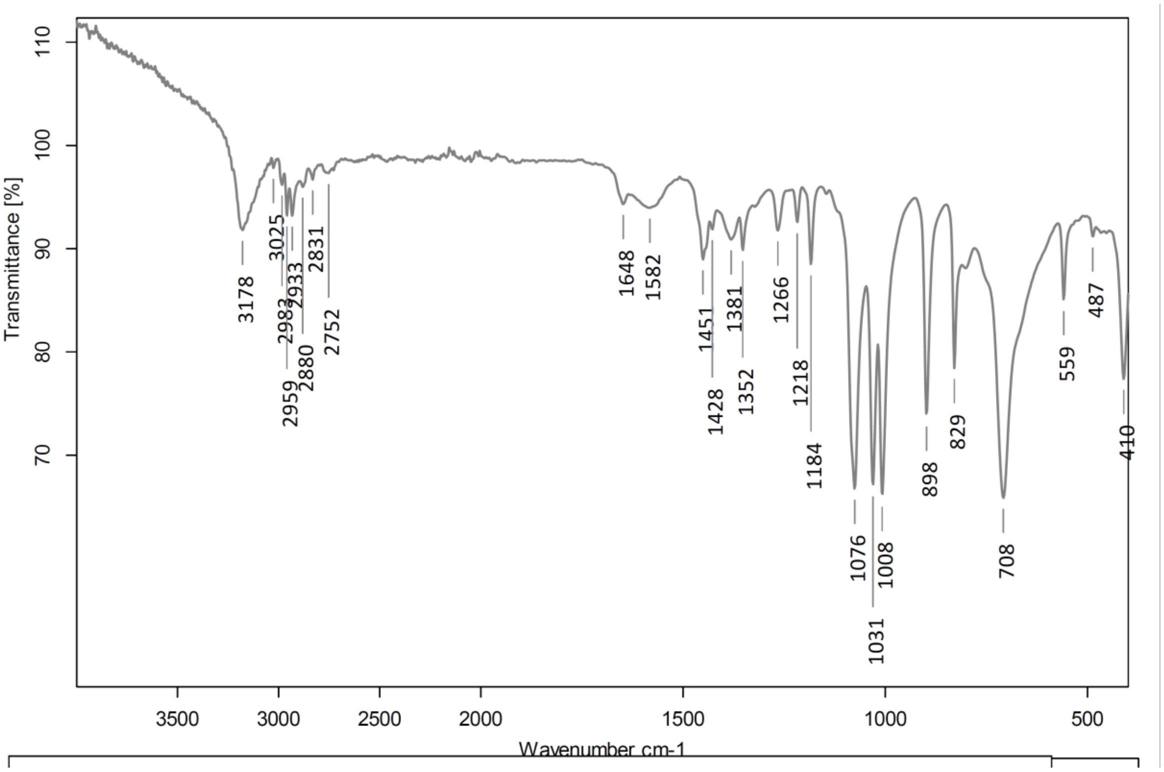

Figure S27. FTIR-ATR spectrum of **14**.

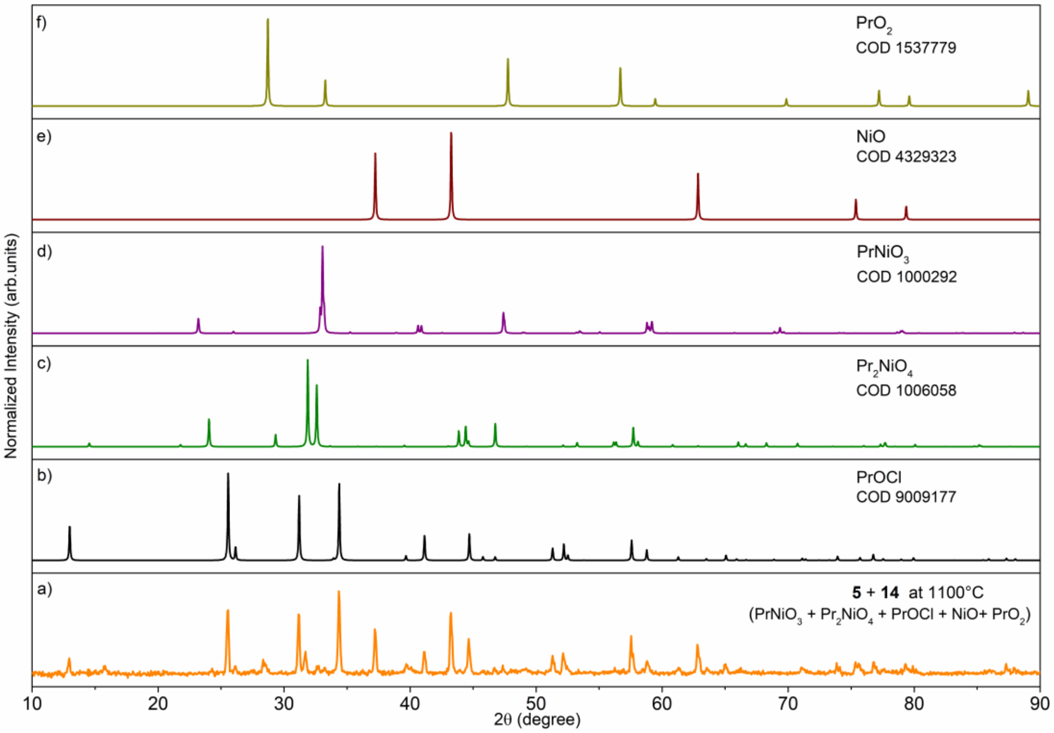

Figure S28. PXRD patterns of oxide materials prepared by calcination of **5** and **14** at 1100 °C (a), PrOCl [9009177] (b), Pr_2NiO_4 [1006058] (c), $PrNiO_3$ [1000292] (d), NiO [4329323] (e), PrO_2 [1537779] (f).

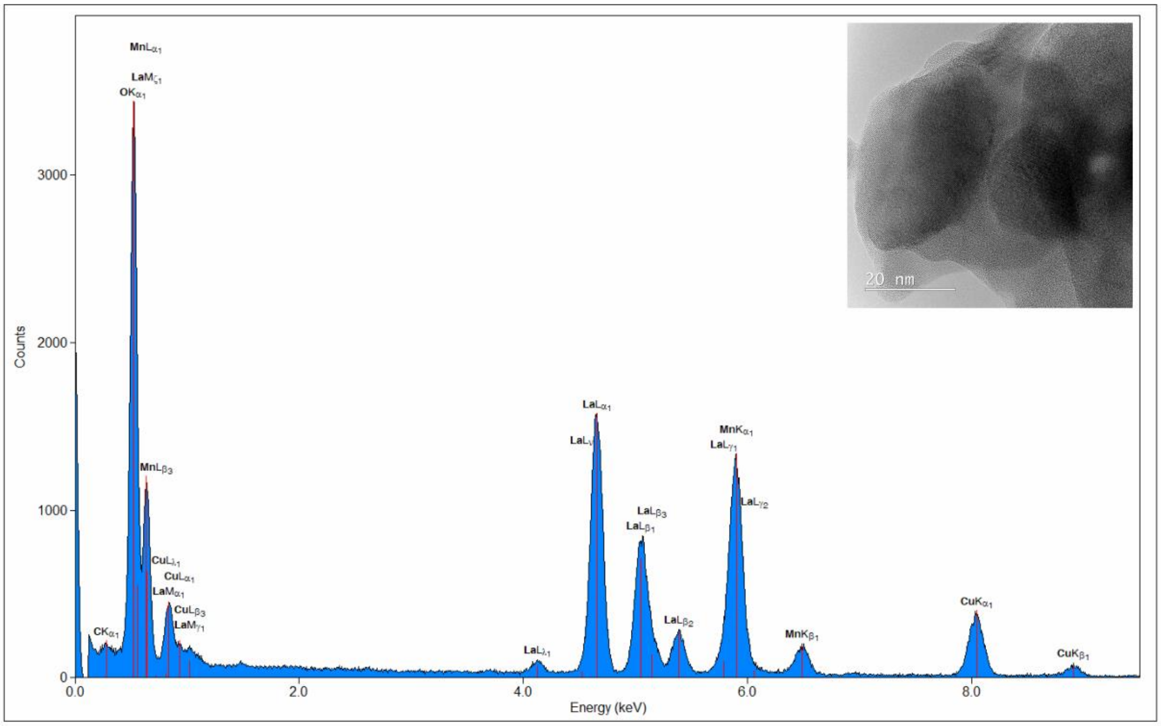

Figure S29. EDS analysis of $LaMnO_3$ prepared by calcination of **1** and **13** at 1100 °C. The copper and carbon elements come from the use of copper carbon grids.

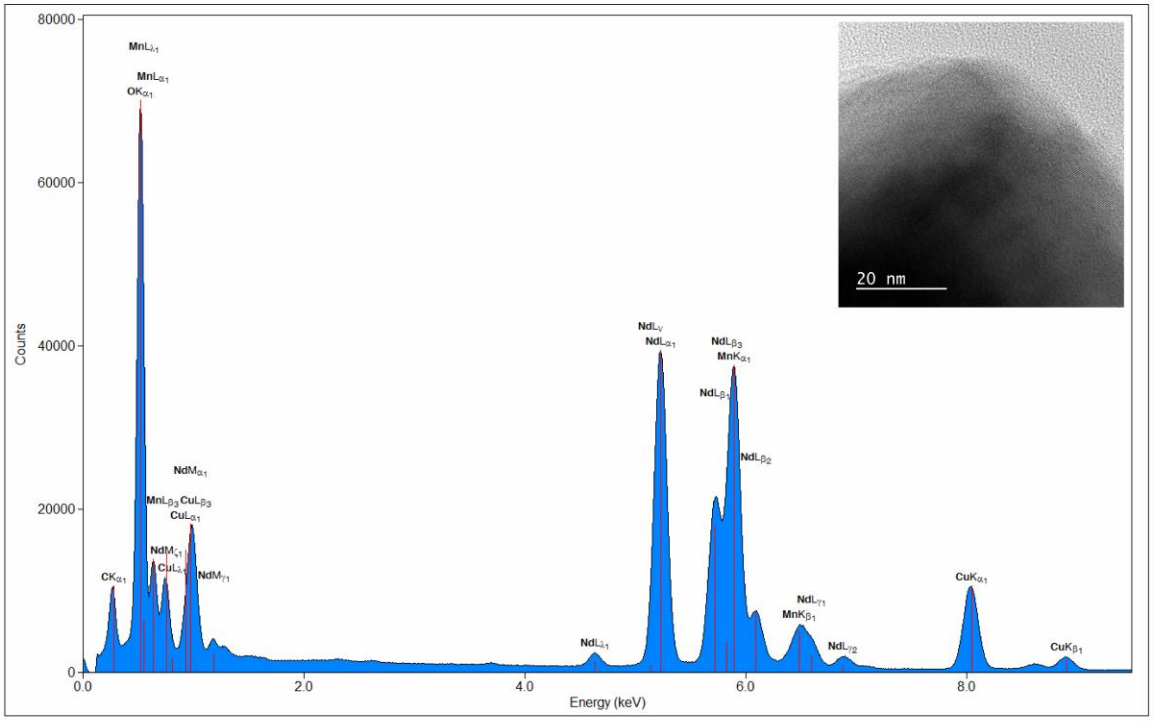

Figure S30. EDS analysis of $NdMnO_3$ prepared by calcination of **2** and **13** at 1100 °C. The copper and carbon elements come from the use of copper carbon grids.

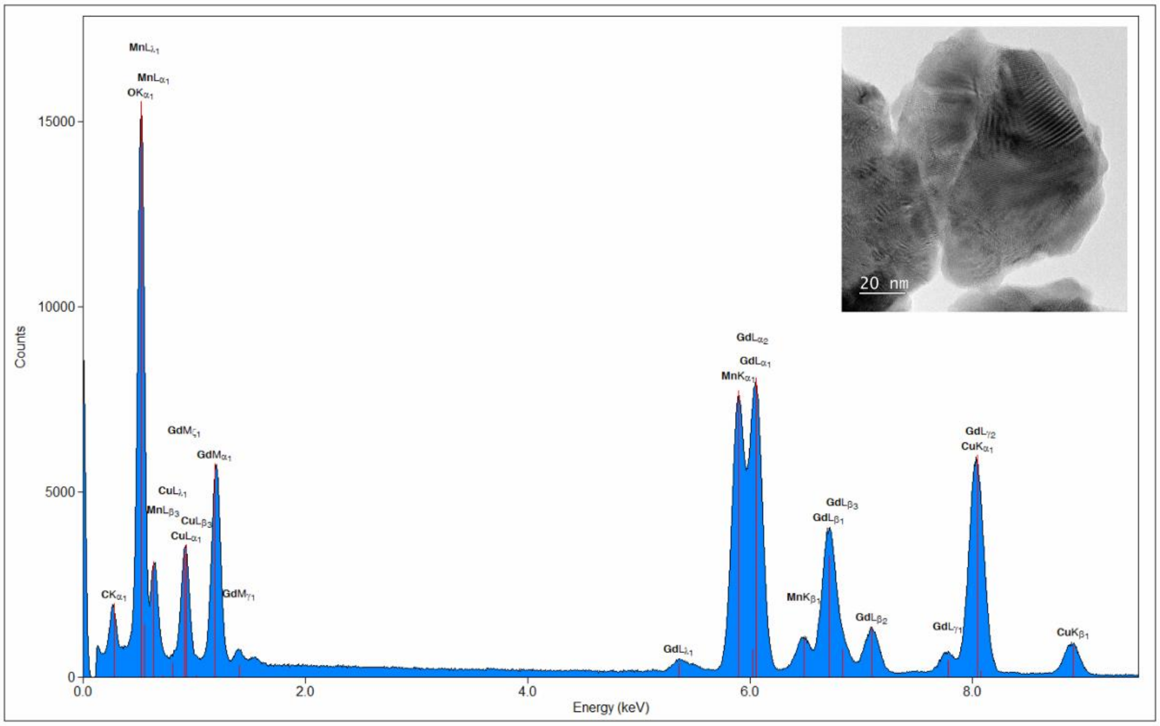

Figure S31. EDS analysis of $GdMnO_3$ prepared by calcination of **3** and **13** at 1100 °C. The copper and carbon elements come from the use of copper carbon grids.

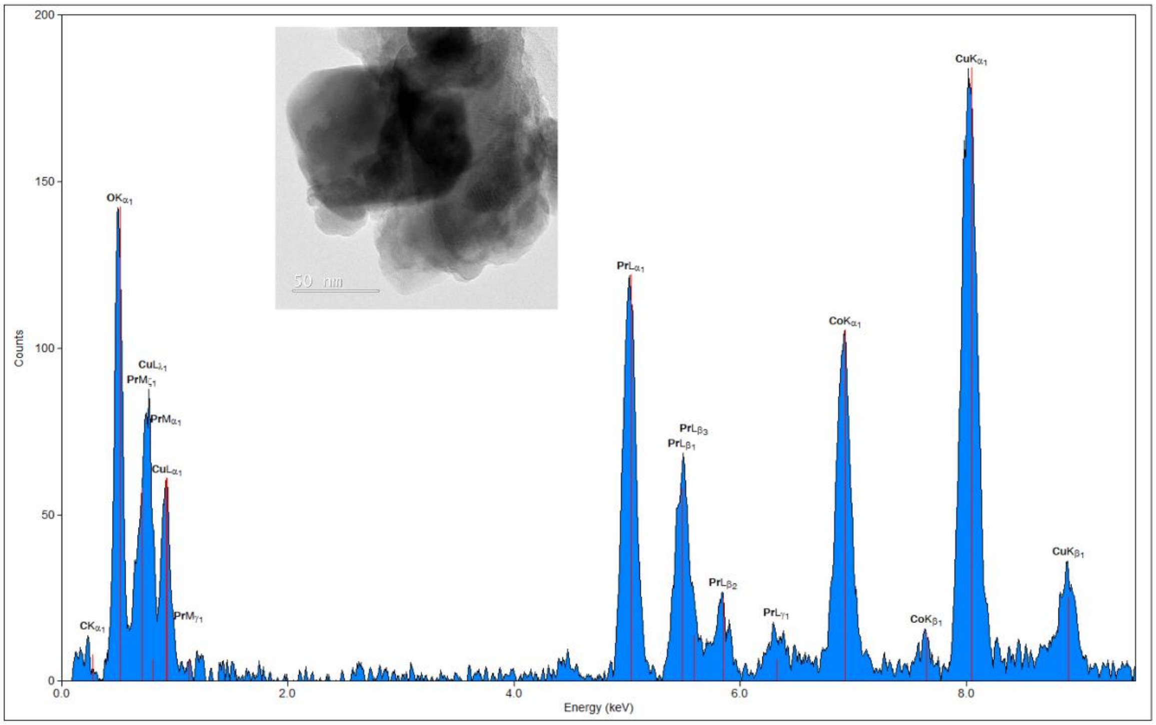

Figure S32. EDS analysis of $PrCoO_3$ prepared by calcination of **4** and **7** at 1100 °C. The copper and carbon elements come from the use of copper carbon grids.

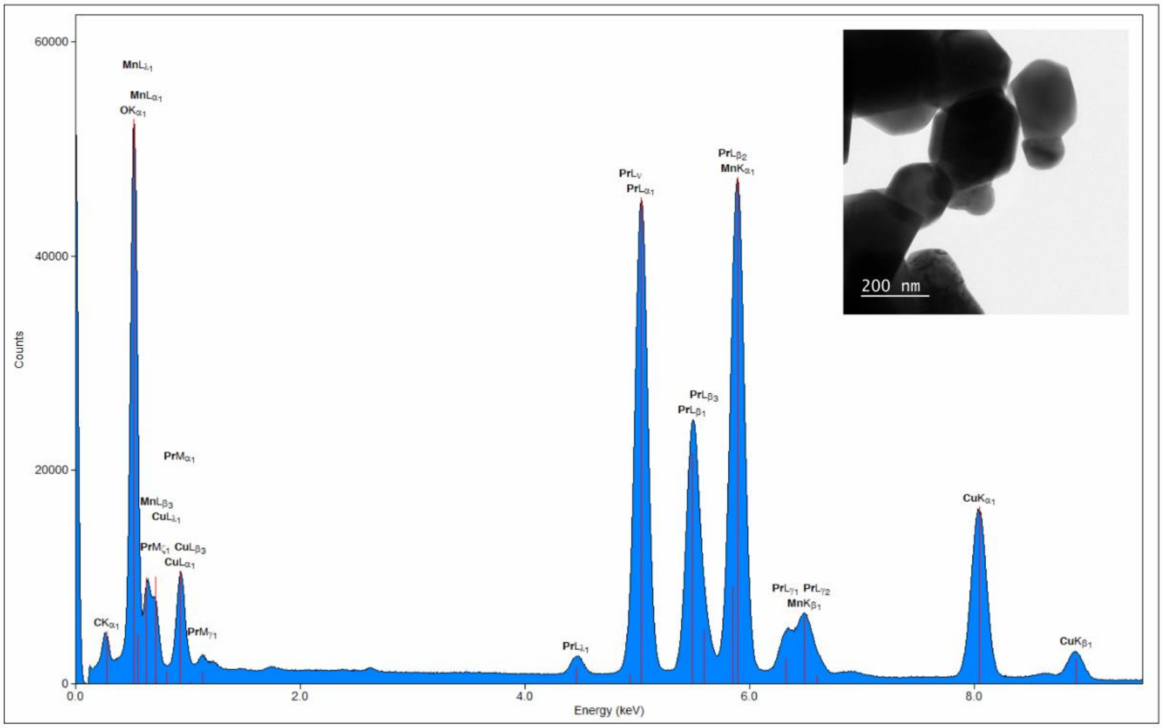

Figure S33. EDS analysis of $Pr_{0.9}MnO_3$ prepared by calcination of **12** and **13** at 1100 °C. The copper and carbon elements come from the use of copper carbon grids.

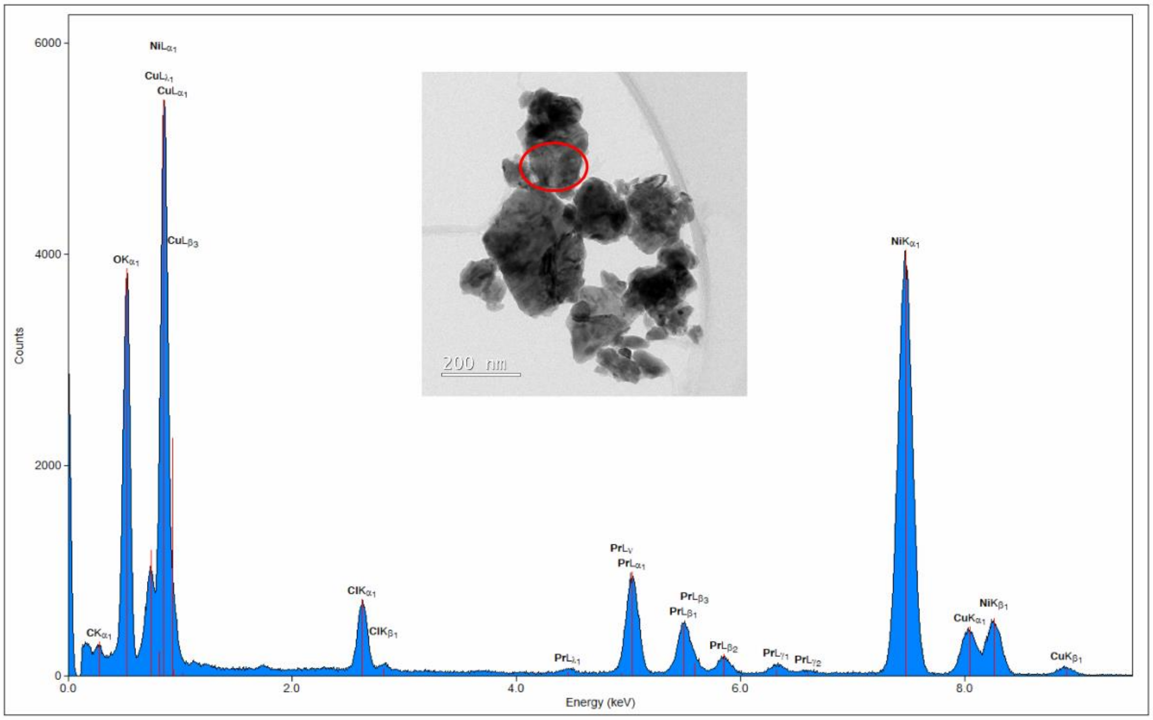

Figure S34. EDS analysis of materials prepared by calcination of **5** at 1100 °C. The copper and carbon elements come from the use of copper carbon grids.

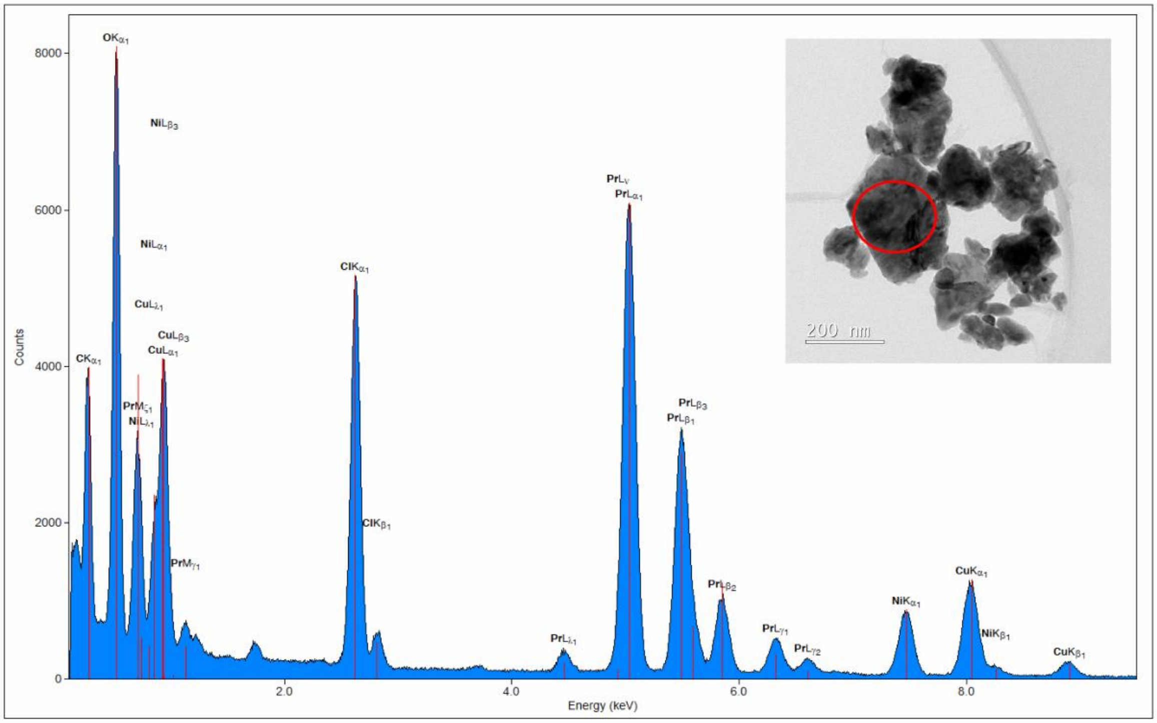

Figure S35. EDS analysis of materials prepared by calcination of **5** at 1100 °C. The copper and carbon elements come from the use of copper carbon grids.

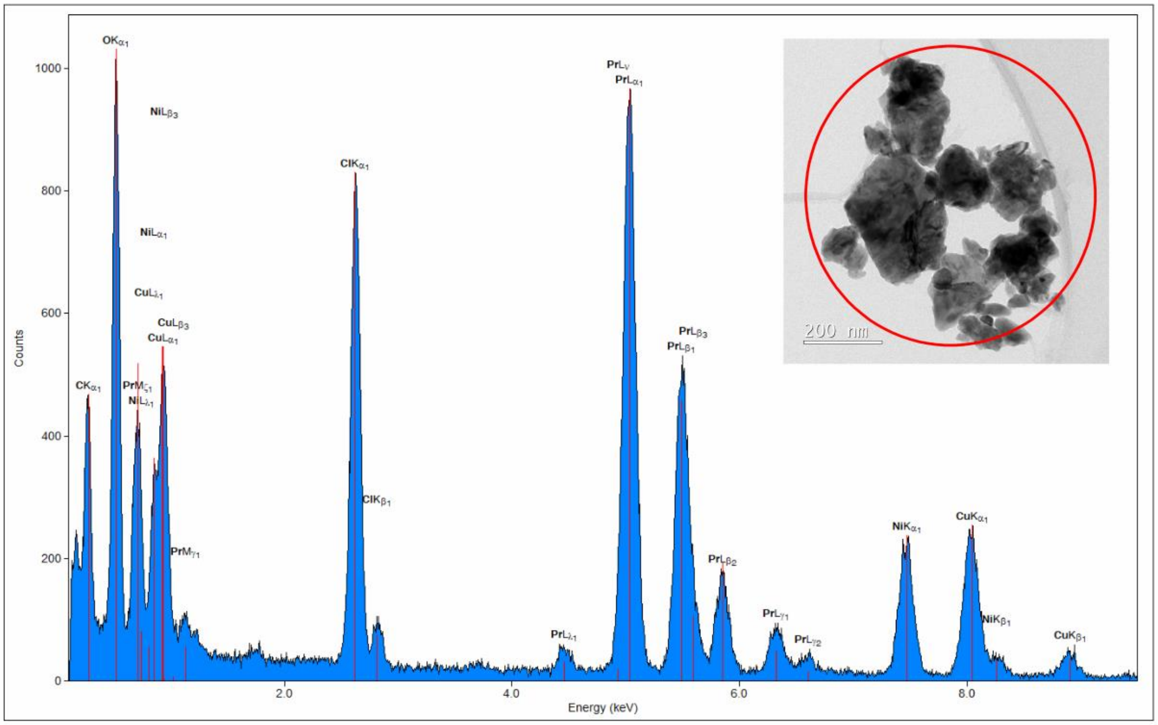

Figure S36. EDS analysis of materials prepared by calcination of **5** at 1100 °C. The copper and carbon elements come from the use of copper carbon grids.
